# Hsa_circ_0001666 suppresses the progression of colorectal cancer through the miR‐576‐5p/PCDH10 axis

**DOI:** 10.1002/ctm2.565

**Published:** 2021-11-04

**Authors:** Jiahui Zhou, Lu Wang, Qingyang Sun, Ranran Chen, Chuan Zhang, Peng Yang, Yuqian Tan, Chaofan Peng, Tuo Wang, Chi Jin, Jiangzhou Ji, Kangpeng Jin, Yueming Sun

**Affiliations:** ^1^ The First School of Clinical Medicine Nanjing Medical University Nanjing China; ^2^ Department of General Surgery The First Affiliated Hospital of Nanjing Medical University Nanjing China

**Keywords:** ceRNA, colorectal cancer, hsa_circ_0001666, miR‐576‐5p, PCDH10

## Abstract

**Background:**

Though circular RNAs, new non‐coding RNA classes have demonstrated that they have an essential role in the initiation as well as development of CRC (colorectal cancer), whereas in CRC the function and mechanism of hsa_circ_0001666 are less known.

**Methods:**

Hsa_circ_0001666 was identified by bioinformatics analysis of a circRNA microarray from the GEO database, and its expression in both CRC cell lines and tissues was analysed. A series of in vitro along with in vivo experiments were carried out for exploring the hsa_circ_0001666 functions, including transwell, wound healing, flow cytometry, colony formation, Edu, CCK‐8, soft agar colony formation, tumor xenografts and lung/liver metastasis in mice. RNA pull‐down, RIP (RNA immunoprecipitation), luciferase reporter assay, FISH (fluorescence in situ hybridization) and rescue experiments were used for determining the correlation among hsa_circ_0001666, miR‐576‐5p and PCDH10.

**Results:**

Hsa_circ_0001666 was downregulated in both CRC cell lines along with tumour tissues. A higher expression level of hsa_circ_0001666 indicated a better clinical prognosis in patients with CRC. Hsa_circ_0001666 knockdown significantly supported CRC cell proliferation along with invasion and inhibited cell apoptosis in vitro. Hsa_circ_0001666 knockdown accelerated the CRC growth and metastasis in vivo. Moreover, the mechanistic study showed that hsa_circ_0001666, acting as ‘ceRNA’ of miR‐576‐5p, prevented PCDH10 downregulation, as well as suppressed EMT and stemness of CRC cells, and the Wnt/β‐catenin signalling pathway. Inhibiting miR‐576‐5p or overexpressing PCDH10 could reverse phenotypic changes caused by knocking down of hsa_circ_0001666.

**Conclusions:**

Hsa_circ_0001666 suppresses CRC progression through the miR‐576‐5p/PCDH10 axis and may provide a new insight for the diagnosis and treatment of CRC.

## Graphical abstract

This study demonstrates that hsa_circ_0001666 suppresses the progression of colorectal cancer.

Hsa_circ_0001666 can regulate the PCDH10 expression by competitively interacting with miR‐576‐5p.

Hsa_circ_0001666 inhibits the Wnt/β‐catenin signalling pathway.

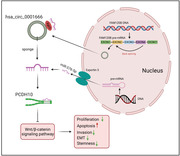

## 1 BACKGROUND

In the whole world, CRC (colorectal cancer) is the third malignancy death cause. Despite advances in screening technology and therapeutics, the estimated CRC deaths were 53,200 in the United States in 2020.^1^, ^2^ Effective treatment strategies for metastatic or recurrent CRC are still deficient, leaving an urgent requirement for discovering an effective and novel molecular target.

CircRNAs (Circular RNAs) are a single‐stranded closed‐loop non‐coding RNA class possessing a canonical splicing junction site without terminal 5′ caps and 3′ poly(A) tails.^3^ In CRC development, recent studies have demonstrated the significant role of circRNAs via multiple biological processes, including tissue differentiation, proliferation, invasion, apoptosis and metastasis.^4^, ^5^, ^6^ Hardly degraded by RNA exonuclease, circRNAs are more stable as compared to linear RNA. Therefore, circRNAs are promising to be novel biomarkers for cancer diagnosis and treatment.^7^, ^8^ In order to explore the novel molecular target of CRC progression, we analysed a microarray dataset in the GEO datasets and found that a circRNA, hsa_circ_0001666, with decreased expression in CRC tissues. Hsa_circ_0001666 was reversely sheared from FAM120B exons 2, 3 and 4. A previous report showed hsa_circ_0001666 could promote tumourigenesis in papillary thyroid carcinoma by upregulating ETV4.^9^ Meanwhile, hsa_circ_0001666 may serve as a potential biomarker of Crohn's disease.^10^ However, there is no report regarding its function in CRC development.

CircRNAs have a variety of functions, including serving as microRNA (miRNA) sponges, interacting with RBPs (RNA‐binding proteins), acting as a transcription factor and assisting in protein translation.^11^, ^12^, ^13^ According to recent studies, circRNAs can function as miRNA sponges by binding competitively to miRNA.^14^, ^15^ For example, circNRIP1 could sponge miR‐629‐3p for promoting invasion and migration of cervical cancer.^16^ Via the miR‐6792‐3p/CAV1 axis, gastric cancer tumour proliferation and metastasis were decelerated by circCCDC9.^4^

PCDH10, a member of the non‐clustered protocadherin subfamily, was reported for suppressing tumour in multiple cancers because of its promoter CpG hypermethylation.^17^, ^18^, ^19^ Functional studies revealed that PCDH10 could inhibit cell proliferation, migration, as well as induce tumour cells apoptosis.^20^, ^21^ Moreover, PCDH10 could suppress epithelial‐mesenchymal transition (EMT) and stemness in CRC by negatively regulating the EGFR/AKT/β‐catenin signalling pathway^22^ and defer the development of endometrial endometrioid carcinoma through the PCDH10–Wnt/β‐catenin–MALAT1 regulatory axis.^23^

Here, we identified a CRC‐related circRNA, hsa_circ_0001666, as a suppressor of CRC progression. Our molecular biology experiments revealed that hsa_circ_0001666 could function as a miR‐576‐5p sponge and regulate PCDH10 expression to modulate the tumourigenesis and metastasis of CRC. Moreover, hsa_circ_0001666 has been expressed as low levels in CRC tissues and related to lower tumour node metastasis (TNM) stage, less lymph node invasion, along with smaller tumour size. Our findings revealed a potential molecule in regulating CRC progression, which may help to refresh the diagnosis and treatment of CRC.

## 2 MATERIALS AND METHODS

### 2.1 CRC tissue samples and cell lines

From 2014 to 2015, 70 colorectal cancer tissues as well as matched normal tissues were collected from patients who had undergone CRC radical resection in The First Affiliated Hospital of Nanjing Medical University.

All tissues were paired in formalin at the same time for wax preparation, and the other was frozen in liquid nitrogen as well as kept at a stable temperature of −80°C until RNA extraction was completed. All clinical and pathological diagnoses were verified by two pathologists in accordance with the eighth edition of American Joint Committee on Cancer (AJCC) and Union for International Cancer Control (UICC). The research was authorized by the Ethics Committee of The First Affiliated Hospital of Nanjing Medical University, and all patients signed an informed consent form.

Human CRC cell lines (LoVo, Caco‐2, DLD‐1, SW480, HCT‐116, SW620, HT‐29) and healthy human colon epithelial mucosa cell line (NCM460) were purchased from the Chinese Academy of Sciences’ Culture Collection Center (Shanghai, China). According to the providers’ instructions, cells were preserved and processed. LoVo, Caco‐2, SW620, SW480, and HEK‐293 T cells were kept in a DMEM medium (Gibco, USA). HT‐29 and HCT‐116 were kept in DMEM/F12 medium (Gibco, USA). DLD‐1 was kept in RPMI‐1640 medium (Gibco, USA). Then, 10% fetal bovine serum (Gibco, USA), 1% penicillin and streptomycin (Gibco, USA) were added to the medium. All cell lines were grown at 37°C in a humidified incubator with 5% CO_2_.

### 2.2 RNA extraction and quantitative real‐time polymerase reaction

TRIzol reagent (Takara, Japan) was utilized for isolating complete RNA from CRC tissues, normal tissues along with cell lines. gDNA (genomic DNA) was extracted using FastPure DNA Isolation (Vazyme, China). Nanodrop 2000 was used to examine the RNA samples’ purity as well as concentration (Thermo Fisher Scientific, USA). mRNA and circRNA were reversely transcribed utilizing the PrimeScript RT master mix (Takara, Japan) and designed primers, with glyceraldehyde‐3‐phosphate dehydrogenase (GAPDH) as an internal control. Reverse transcriptions for miRNA were carried out utilizing Bulge‐Loop™ miRNA quantitative real‐time polymerase reaction (qRT‐PCR) starter kit (RIBOBIO, China) and unique stem‐loop primers, with U6 as an internal control. cDNA was amplified with the ABI Prism 7500 sequence detection system and TB Green Premix Ex Taq II (Takara, Japan) (Applied Biosystems, USA). These procedures were carried out for each sample in triplicate. The 2^−ΔΔ^ *^CT^* technique was utilized for quantifying the circRNA, miRNA, and mRNA expression levels with the internal regulation. The back‐splice junction of circRNA was detected using divergent primers, while linear mRNA was detected using convergent primers. The primers are listed in Table S1 the Supporting Information.

### 2.3 RNase R treatment

RNase R (3 U/g, Epicenter) was used to treat RNAs (10 μg) from LoVo and DLD‐1 cells, followed by incubation at 37°C for 30 min. Afterwards, circRNA and linear RNA were detected using reverse transcription and qRT‐PCR.

### 2.4 Nucleic acid electrophoresis

To analyse the gDNA and cDNA PCR results, we employed agarose gel electrophoresis (2%) using 45 mmol/L Tris‐boric acid; 1mmol/L EDTA (TBE) as the running buffer. DNA was also isolated by electrophoresis at 120 V for 30 min at room temperature. Marker L (50‐500 bp) was used as a DNA marker (Beyotime, China). UV irradiation was used to examine the bands.

### 2.5 siRNA, shRNA, miR‐mimics and miR‐inhibitor transfection

Before transfection, LoVo and DLD‐1 cells were seeded in six‐well plates as well as grown to 70%–80% confluence. Si‐hsa_circ_0001666(1‐3), miR‐576‐5p mimics, miR‐576‐5p inhibitor, si‐PCDH10 and negative control oligonucleotides were designed as well as synthesized by RIBOBIO (Guangzhou, China). Lipofectamine3000™ reagent (Invitrogen, USA) was utilized for transfection following the manufacturer's protocol. Human lentivirus‐sh‐hsa_circ_0001666 and lentivirus‐oe‐hsa_circ_0001666 as well as negative control lentivirus were packed and purchased from Genomeditech (Shanghai, China). Cells were transfected following manufacturer's protocol.

### 2.6 Vector construction

The full‐length cDNAs of hsa_circ_0001666 and PCDH10 were synthesized as well as cloned into overexpression vector pLCDH‐ciR to create an overexpression vector. An empty vector with no hsa_circ_0001666 or PCDH10 sequence was utilized as the negative control.

### 2.7 Luciferase reporter assay

The hsa_circ_0001666 and PCDH10–3′UTR sequences, as well as their corresponding mutations, were synthesized, designed, transfected into luciferase reporter vector pmiR‐RB‐Report vector (RIBOBIO, China), and then designated as hsa_circ_0001666‐WT, hsa_circ_0001666‐MUT, PCDH10–3′UTR‐WT, along with PCDH10–3′UTR‐MUT. In this study, all of these plasmids were co‐transfected into HEK‐293T cells together with miR‐576‐5p mimics and miR‐576‐5p mimics NC. Following that, the relative luciferase activity was determined utilizing the dual luciferase assay kit (Promega, USA) that was performed as per the instructions of the manufacturer.

### 2.8 Biotin‐coupled probe RNA pull‐down assay

Pull‐down assays with biotinylated hsa_circ_0001666 (RIBOBIO, China) were carried out. A total of 1 × 10^7^ CRC cells were sonicated and lysed. Probe‐coated beads were produced by incubation with probes—Dynabeads M‐280 streptavidin (Invitrogen, USA) for 2 h at 25°C. The cell lysates were incubated overnight at 4°C. After being washed with the wash buffer, the RNA complexes bound to the beads were eluted as well as purified using TRIzol Reagent (Takara, Japan).

### 2.9 RNA immunoprecipitation

The RNA immunoprecipitation (RIP) assay was performed as per the instructions of the manufacturer using the immunoprecipitation kit for the Magna RIP™ RNA protein (Millipore, USA). LoVo cells were lysed into complete RIP buffer after transfection with miR‐576‐5p mimic or negative control. The cell extract were subsequently combined with an anti‐argonaute 2 (AGO2) or anti‐IgG antibody at a magnetic perforation of 4°C at 6 h (Millipore, USA). The beads were washed as well as treated with Proteinase K for the extraction of proteins. Finally, TRIzol Reagant (Takara, Japan) was used to extract isolated RNA, followed by a qRT‐PCR analysis of the purified RNA.

### 2.10 Fluorescence in situ hybridization

In CRC cells, the location of hsa_circ_0001666 as well as miR‐576‐5p has been determined using the fluorescence in situ hybridization (FISH) assay. Cy3‐labelled hsa_circ_0001666 probes as well as Carboxyfluorescein (FAM)‐labelled miR‐576‐5p were designed and purchased from RIBOBIO (Guangzhou, China). Hsa_circ_0001666 and miR‐576‐5p probes were hybridized overnight as per the instructions of the manufacturer. A C2+ confocal microscope was utilized for capturing the images (Nikon, Japan).

### 2.11 Cell counting kit‐8 proliferation assay

In each well of a 96‐well plate, 1000 cells were placed. The CCK‐8 reagent (Dojindo Seed, Japan) was added to the culture medium directly at the stated time (24, 48, 72, 96 h). After that, cells were incubated for 2 h at 37°C, and the OD (optional density) value was calculated at 450 nm by a microplate reader (BioTek Instruments, USA). The procedure was carried out three times.

### 2.12 Colony formation assay

One thousand cells were seeded in each well of the six‐well plates, whereas for 2 weeks it was cultured in a humidified incubator with 5% CO_2_. The cells were then washed in PBS (phosphate‐buffered saline), fixed with 4% paraformaldehyde for 10 min as well as stained for another 20 min with a 0.5% crystal violet solution. After that, the colonies were counted and examined. The assay was carried out three times.

### 2.13 5‐Ethynyl‐2'‐deoxyuridine incorporation assay

BeyoClick™ EdU‐555 Cell Proliferation Kit (Beyotime, China) was used after following the manufacturer's protocol to determine cell proliferation. A total of 3 × 10^4^ cells were cultured for 24 h in 96‐well plates. After a 2‐ h incubation with 20 μM 5‐ethynyl‐2'‐deoxyuridine (EdU) solution, both cell lines were fixed with 4% paraformaldehyde then secured using Apollo Dye Solution and DAPI. Cells were then photographed and counted under a C2+ confocal microscope (Nikon, Japan). The assay was carried out three times.

### 2.14 Wound healing assay

In total 1 × 10^6^ cells were seeded throughout 24 h in six‐well plates. A 200‐μL pipette blade was then scraped onto the monolayer. At 0 and 48 h after injury, 10 high‐power fields were photographed to capture representative images of cell migration. The diminishing distance across the induced injury region was measured as well as expressed as a relative migration rate, normalized to the 0 h power. The assay was performed at least three times with three replicates for each one.

### 2.15 Transwell migration and invasion assay

A total of 3 × 10^5^ cells in the upper chamber were seeded in the transwell test with 200 μL of a serum‐free medium. The transwell chamber (Corning, USA) was coated for invasion tests and without matrigel mix for migration tests by Matrigel mix (BD Biosciences, USA). The medium in the lower chamber had 10% of FBS, which might attract cells. The cells were stained with a solution of crystal violet 0.5% for 20 min after incubation lasting 48 h. The cell lines were photographed and counted in five distinct regions. The assay was carried out three times.

### 2.16 Soft agar colony formation assay

Taken together in the semi‐solid agar medium, 2.5 × 10^4^ cells were insulated into each well (0.5% agarose/PBS culture medium on the six‐well plate at 0.6% agarose/PBS base). Representative cell colonies images were collected using an inverted light microscope after 0, 7 or 14 days of incubation (Zeiss, Primovert).

### 2.17 Flow cytometry assay

The Annexin V‐FITC/ PI kit was utilized for assessing apoptosis (Vazyme, Nanjing, China). These cells were trypsinized, washed with ice‐cold PBS and stained with Annexin V‐FITC/PI. The cells were analysed using a flow cytometer after 20‐min incubation (BD FACSCANTO II, BD Biosciences, San Jose, CA, USA).

Cells were washed two times in PBS as well as centrifuged for 5 min at 350 × *g* for an examination of the percentage of CD44 in the cells treated. Then cells were resuspended with an anticorps of 1 g/mL (BD Pharmingen, Franklin Lakes, NJ, USA) in 2 mL surface staining buffer (PBS, pH 7.4, 0.1% BSA) with antibodies of 4°C for 20 min. The cells were therefore replaced with PBS without washing and tested according to the recommendations of the factory with a FACS flow cytometer. All data were evaluated with CytExpert software.

### 2.18 Western blot

Cells were lysed with the RIPA (radio immunoprecipitation assay, Beyotime, China). By using bicinchoninic acid (BCA) analysis, the protein was prepared and quantified (Beyotime, China). Similar protein quantity was extracted using 10% SDS–PAGE as well as transferred to polyvinylidene difluoride membranes in the same way (Millipore, MA, USA) and blocked using 5% skim milk powder. Then the blocked protein was incubated with primary antibody anti‐PCDH10 (1:1000, Proteintech, Wuhan, China), anti‐β‐catenin (1:10,000, Proteintech, Wuhan, China), anti‐LEF1 (1:1000, Cell Signaling Technology, MA, USA), anti‐cyclin D1 (1:1000, Cell Signaling Technology, MA, USA), anti‐CD133 (1: 1000, Cell Signaling Technology, MA, USA), and anti‐SOX2 (1:1000, Cell Signaling Technology, Danvers, MA, USA), anti‐CD44 (1:1000, Cell Signaling Technology, MA, USA), anti‐E‐cadherin (1:1000, Cell Signaling Technology, MA, USA), anti‐N‐cadherin (1:1000, Cell Signaling Technology, MA, USA), anti‐Vimentin (1:1000, Cell Signaling Technology, MA, USA), anti‐Snail (1:1000, Cell Signaling Technology, MA, USA) and anti‐GAPDH (1:5000, Cell Signaling Technology, MA, USA) at 4°C overnight. After that, the membranes were incubated with a secondary antibody (1:5000, Cell Signaling Technology, MA, USA) for 2 h. Finally, Omni‐ECL chemiluminescent reagent (EpiZyme, China) was used to visualize the blots, and the results were analysed using Image Lab software.

### 2.19 Immunohistochemistry examination

Tissue samples were embedded in paraffin, fixed in 4% paraformaldehyde as well as sectioned. Anti‐Ki‐67 primary antibodies were incubated overnight at 4°C on the tissue before incubation with an HRP‐conjugated secondary antibody.

### 2.20 Tumour xenografts and lung/liver metastasis in mice

Animal experiments were authorized by the Animal Care Committee of the Nanjing Medical University and conducted in accordance with National Health Institutes. The influence of hsa_circ_0001666 on tumour development was researched using 5‐week‐old female BALB/c nude mice. LoVo cells (1 × 10^6^, 100 μL) stably transfected with sh‐hsa_circ_0001666 or negative control and DLD‐1 cells stably transfected with oe‐hsa_circ_0001666 or control vector were injected subcutaneously into the armpit of nude mice in a tumour growth assay. The tumour volume (length × width^2^/2) was calculated by a caliper on a weekly basis. Finally, the mice were sacrificed, whereas subcutaneous tumour tissue were examined using immunohistochemistry (IHC) staining with anti‐Ki‐67.

Sh‐hsa_circ_0001666 or NC LoVo cells, as well as oe‐hsa_circ_0001666 or control vector DLD‐1 cells (1 × 10^6^), were inserted into the ileocolic vein of nude mice to study tumour lung metastasis. To assess tumour liver metastasis, the upper left lateral abdomen was incised to expose the spleen, then stably transfected LoVo and DLD‐1 cells (1 × 10^6^) were injected into the spleen's distal tip. Lungs as well as livers were removed after 30 days, paraffin‐embedded and, eventually, the metastasis was confirmed with H&E (hematoxylin and eosin staining).

### 2.21 Statistical analysis

SPSS 22.0 (IBM, SPSS, Chicago, USA) and GraphPad Prism 8 were used for statistical analysis. To explore whether two or more classes were statistically significant, we utilized one‐way analysis of variance and Student's *t*‐test. The relationships were examined using the Pearson correlation coefficient. The data were interpreted as a mean standard deviation. The Kaplan–Meier technique was utilized for measuring OS curves, which were then evaluated using the log‐rank test. *p* < .05 were statistically significant for all results.

## 3 RESULTS

### 3.1 Hsa_circ_0001666 is lowly expressed in CRC tissues and cell lines and mainly localized in the cytoplasm

**FIGURE 1:**
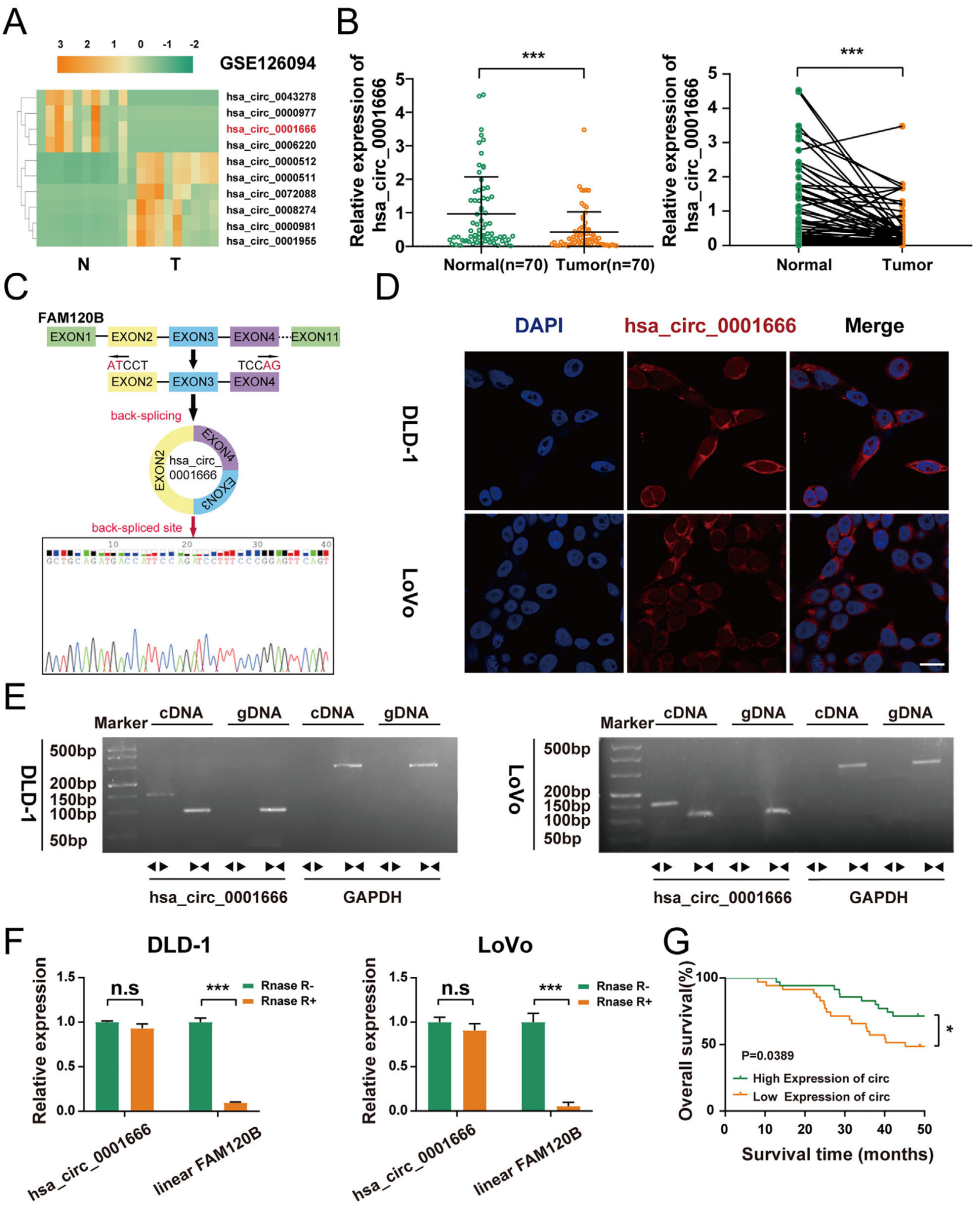
Hsa_circ_0001666 is lowly expressed in CRC tissues and cell lines and mainly localized in the cytoplasm. (A) Heatmap based on GSE126094, four most downregulated and six most upregulated circRNAs (adjusted *p*<.05 and log|FC|>4). (B) qRT‐PCR was used to detect the relative expression level of hsa_circ_0001666 in CRC tissues and matched para‐cancer tissues (*n* = 70). (C) The schematic illustration showed the FAM120B exon two to four circularization into hsa_circ_0001666; Sanger sequencing showed the head‐to‐tail splicing of hsa_circ_0001666. (D) The FISH assay revealed that hsa_circ_0001666 was mainly found in the cytoplasm. The nuclei were dyed blue with DAPI, and the hsa_circ_0001666 was stained red (scale bar, 100 μm; magnification, 600×). (E) RT‐PCR confirmed the existence of hsa_circ_0001666 both in DLD‐1 and LoVo. Divergent primers amplified hsa_circ_0001666 in cDNA but not in gDNA. GAPDH was used as a negative control. (F) The expression of hsa_circ_0001666 and FAM120B mRNA in both DLD‐1 and LoVo cell lines was detected by qRT‐PCR with or without RNase R. (G) Kaplan–Meier survival curves of CRC patients with low and high hsa_circ_0001666 expression. The median value was used as a cutoff. Data were all showed as mean ± SD (*n* = 3); n.s indicated no significance, *p < .05, ***p < .001

We analysed the dataset GSE126094 in the GEO datasets, which contained 10 pairs of CRC as well as their corresponding paracancerous tissues. Based on the criterion of adjusted *p*‐value<.05 and log| FC |>4 (analysed by GEO2R), six upregulated circRNAs along with four downregulated circRNAs were discovered (Figure 1A). Given that the downregulated circRNAs were promising to function in ncRNA replacement therapies,^24^ we detected the expression levels of four downregulated circRNAs (hsa_circ_0006220, hsa_circ_0001666, hsa_circ_0000977, hsa_circ_0043278) in 70 frozen CRC tissues along with their matched paracancerous tissues through qRT‐PCR. These outcomes illustrated that hsa_circ_0001666 was the most significantly downregulated circRNA in the tumour group (Figure 1B; Figure S1A–C in the Supporting Information) (*p*<.05). Hsa_circ_0001666 has thus been selected for the following investigation.

Hsa_circ_0001666, derived from the FAM120B gene on chromosome 6, consists of exon2, exon3, and exon4 spliced head to tail. Sanger sequencing showed that the back‐spliced sequence of hsa_circ_0001666 was consistent with that in circBase (Figure 1C). Next, FISH assay was carried out and results showed that hsa_circ_0001666 was localized predominately in the cytoplasm, suggesting its key role in post‐transcriptional processes (Figure 1D). Then, to rule out genomic rearrangement of the host gene, convergent primers for FAM120B mRNA and divergent primers for hsa_circ_0001666 were built. cDNA and gDNA were isolated from DLD‐1 and LoVo cells and analysed with agarose gel electrophoresis. We found hsa_circ_0001666 was only detected in cDNA (Figure 1E). For confirming the stability of circRNA, DLD‐1 and LoVo cells were treated separately with RNase R and the relative expressions of hsa_circ_0001666 and linear FAM120B were detected by qRT‐PCR. It is also observed that hsa_circ_0001666 was more stable than linear FAM120B (Figure 1F) (*p*<.001).

Furthermore, based on the clinical data extracted from the 70 CRC tissues, a higher hsa_circ_0001666 expression level was related to lower TNM stage, less lymph node invasion as well as smaller tumour size (Table 1). Moreover, survival information of these patients was gathered and the overall survival (OS) curves have been drawn with the Kaplan–Meier approach. Patients with a higher hsa_circ_0001666 expression level in their CRC tissues had a higher overall survival rate (*p* = .0389) (Figure 1G). Overall, hsa_circ_0001666 was discovered to be a significant prognostic marker that was worthy of further investigation.

**TABLE 1:** Correlation between expression of hsa_circ_0001666 and clinicopathological features in 70 fresh‐frozen CRC tissues

		hsa_circ_0001666 expression		
Characteristics	Case	Low	High	*p* value
All cases	70	49	21	
Age at surgery (years)				.792
<60	25	17	8	
≥60	45	32	13	
Gender				.794
Male	42	30	12	
Female	28	19	9	
Tumour size (cm)				.001*
≥5	40	35	5	
<5	30	14	16	
T grade				.285
T1+T2	26	16	10	
T3+T4	44	33	11	
Lymph node invasion				.035*
Negative (N0)	39	23	16	
Positive (N1–N3)	31	26	5	
Tumour site				.421
Cardiac	7	4	3	
Non‐cardiac	63	45	18	
TNM stage				.040*
I–II	37	21	16	
III–IV	33	28	5	

* *p*<.05.

### 3.2 Hsa_circ_0001666 suppresses the proliferation and invasion and induces the apoptosis of CRC cells in vitro

Hsa_circ_0001666 was lowly expressed in CRC cell lines compared with normal colon epithelial cell line (NCM460) (Figure S2A in the Supporting Information) (*p* < .01). The highest and lowest hsa_circ_0001666 expression levels were found in LoVo and DLD‐1, respectively. For analysing the hsa_circ_0001666 functions, these two cell lines were chosen for experiments.

Three siRNAs against hsa_circ_0001666 were transfected into LoVo cells. Si‐hsa_circ_0001666‐1 had the highest inhibitory efficiency. Also, the overexpression vector of hsa_circ_0001666 was constructed in DLD‐1 cells and achieved a high overexpression efficiency. Meanwhile, the expression of FAM120B mRNA was not changed (Figure S2B,C in the Supporting Information) (*p*<.01).

**FIGURE 2:**
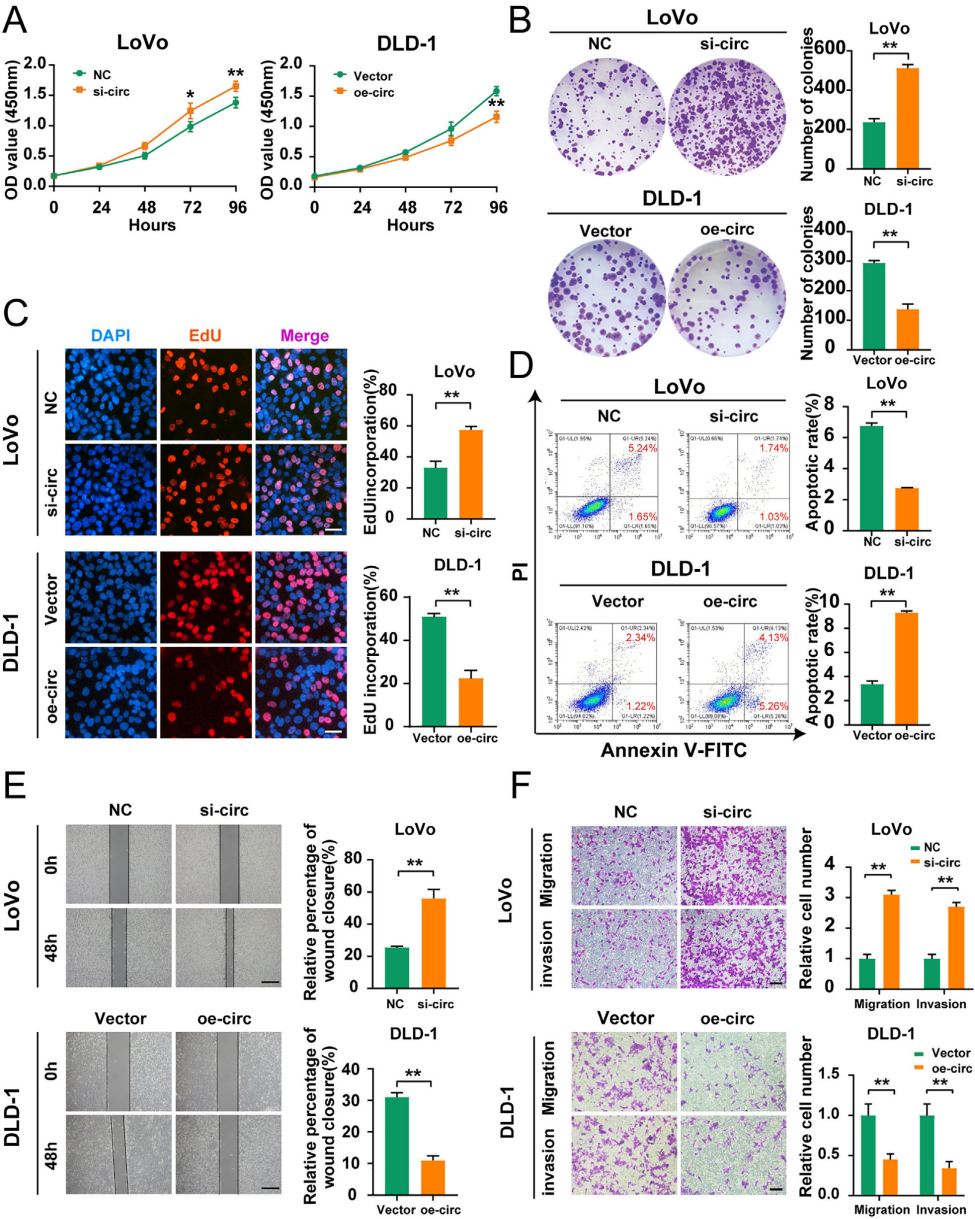
Hsa_circ_0001666 suppresses the proliferation, invasion and induces the apoptosis of CRC cells in vitro. LoVo cells were transfected with si‐circ or NC, and DLD‐1 cells were transfected with oe‐circ or Vector. (A–C) To evaluate the proliferative ability, CCK‐8 assays, colony formation assays and EdU assays were used (magnification, 200×; scale bar, 100 μm). (D) The Annexin‐V FITC/PI staining was used to assess apoptotic rates. (E. F) Wound healing assays and transwell assays were used to evaluate the migratory and invasive capabilities. The scale bar in wound healing assays indicated 20 μm; the scale bar in transwell assays indicated 200 μm. Data were all represented as mean ± SD (*n* = 3). *p < .05, **p < .01

Downregulation of hsa_circ_0001666 substantially increased the proliferative viability of cells in CCK‐8 assays, while upregulation of hsa_circ_0001666 showed the opposite impact (Figure 2A) (*p*<.05). Colony formation assays also showed that downregulating hsa_circ_0001666 significantly enhanced the cloning ability of LoVo cells, whereas upregulating hsa_circ_0001666 greatly harmed this ability (Figure 2B) (*p*<.01). Similarly, in EdU assays, hsa_circ_0001666 knockdown increased the percentage of EdU‐positive cells, whereas hsa_circ_0001666 upregulation decreased the percentage (Figure 2C) (*p*<.01). These findings indicated that hsa_circ_0001666 inhibited CRC cell proliferation.

The apoptosis assays suggested the apoptotic rate in LoVo cells was reduced following the silence of hsa_circ_0001666, while it rose in DLD‐1 cells with overexpressed hsa_circ_0001666 (Figure 2D) (*p*<.01). This experiment recommended that hsa_circ_0001666 promoted the CRC cells’ apoptosis.

Finally, we performed wound healing and transwell assays for evaluating the hsa_circ_0001666 effects on invasion and migration. The findings revealed that the downregulation of hsa_circ_0001666 in LoVo significantly improved the migratory and invasive abilities of CRC cells, while its upregulation in DLD‐1 substantially suppressed these changes (Figure 2E,F) (*p*<.01). These findings suggested that hsa_circ_0001666 inhibited CRC cell invasion and migration.

### 3.3 Hsa_circ_0001666 suppresses the proliferation and metastasis of CRC cells in vivo

**FIGURE 3:**
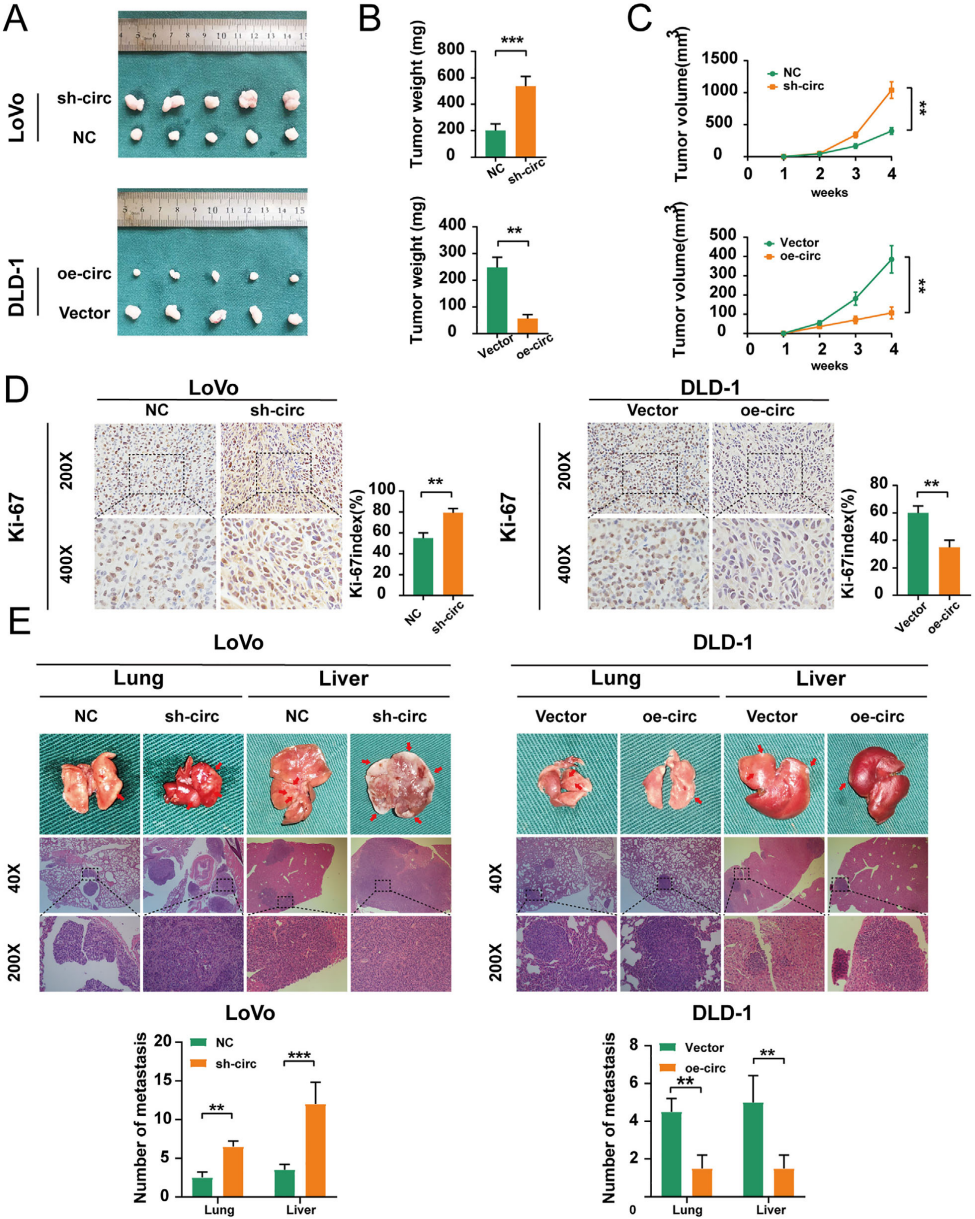
Hsa_circ_0001666 suppresses the proliferation and metastasis of CRC cells in vivo. (A–C) 1 × 10^6^ treated LoVo and DLD‐1 cells were injected subcutaneously into nude mice. After a month, the tumours were dissected and photographed. Tumour volume was measured as (length × width^2^)/2. (D) IHC revealed relative Ki‐67 expression levels in subcutaneous tumour tissues. (E) 1 × 10^6^ treated LoVo cells and DLD‐1 cells were, respectively, injected into the spleen and ileocolic vein of nude mice. Mice were then sacrificed and the lungs, and livers were stained by H&E. The number of metastases was calculated. Data were represented as mean ± SD (*n* = 3). **p < .01. ***p < .001

In order to confirm hsa_circ_0001666 effects on tumour proliferation, we transfected the lentivirus‐sh‐hsa_circ_0001666 and lentivirus‐oe‐hsa_circ_0001666 into LoVo and DLD‐1 cells. We achieved satisfactory transfection efficiency (Figure S2D,E in the Supporting Information) (*p* < .001). LoVo‐NC/sh‐circ and DLD‐1‐Vector/oe‐circ were injected subcutaneously into BALB/c naked mice. The tumour volume was measured per week, and tumour weight was estimated 1 month later. The mouse model showed that hsa_circ_0001666 downregulation caused a significant increase in tumour volume and weight, while hsa_circ_0001666 upregulation reversed this change (Figure 3A–C) (*p*<.01). IHC assays on these subcutaneous tumour tissues showed that the Ki‐67 index was upregulated in the sh‐hsa_circ_0001666 group and downregulated in the oe‐hsa_circ_0001666 group (Figure 3D) (*p*<.01). Then pretreated LoVo cells and DLD‐1 cells were, respectively, injected into the spleen and the ileocolic vein for investigating the function of tumour metastasis. We found more metastatic nodules in the liver and lungs in the sh‐circ group, but much less in the oe‐circ group (Figure 3E) (*p*<.01). Taken together, these experiments showed that hsa_circ_0001666 suppressed the CRC cells’ proliferation along with metastasis in vivo.

### 3.4 Hsa_circ_0001666 binds to miR‐576‐5p directly in CRC cells

**FIGURE 4:**
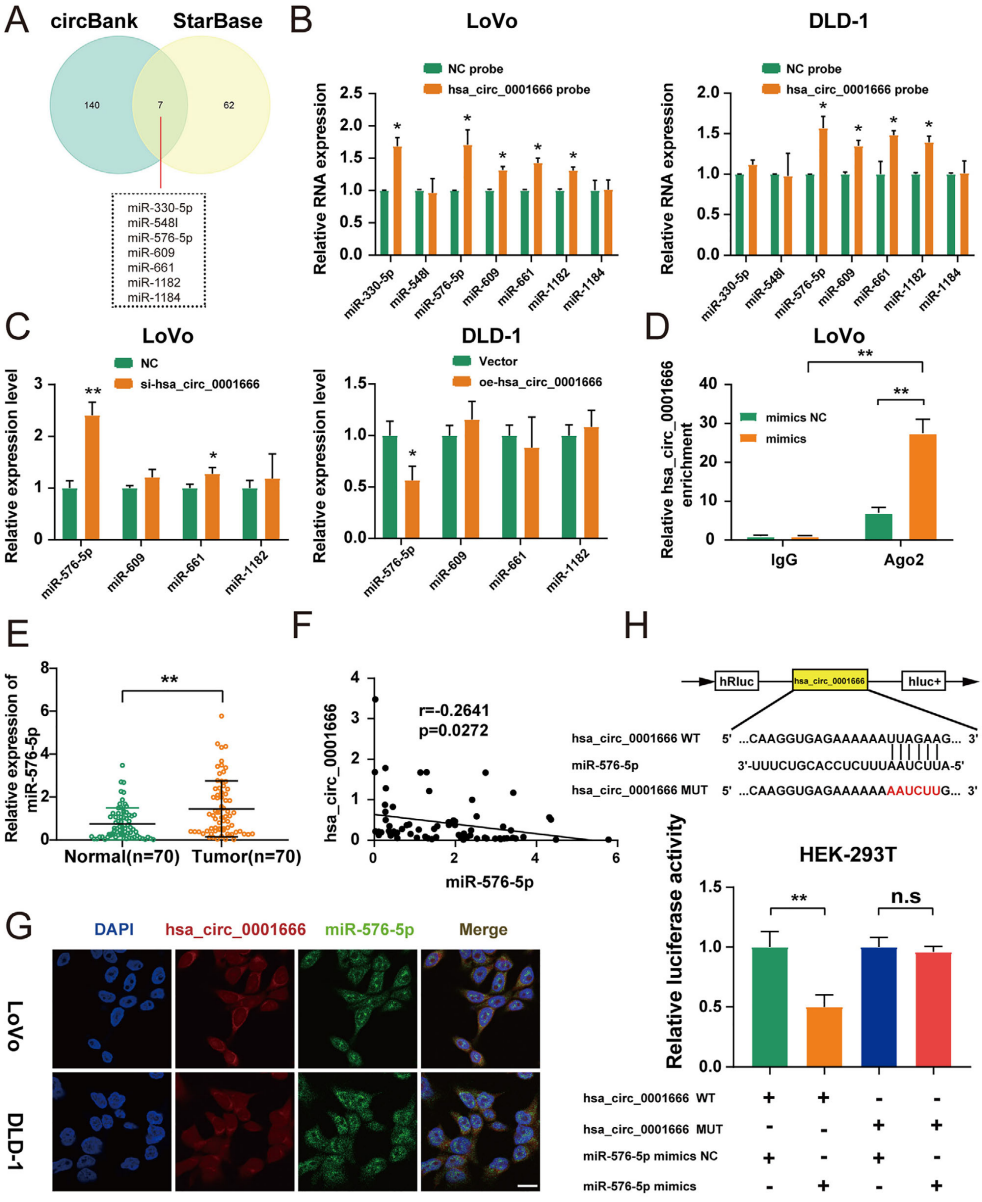
Hsa_circ_0001666 binds to miR‐576‐5p directly in CRC cells. (A) Schematic illustration exhibited miRNAs of hsa_circ_0001666, based on circBank and StarBase. (B) Biotinylated hsa_circ_0001666 probe and an NC probe were used to pull down the candidate miRNAs in both LoVo and DLD‐1 cell lines, and expression levels were tested with quantitative real‐time PCR. The relative level of hsa_circ_0001666 was normalized to the level of NC. (C) Relative expression levels of four candidate miRNAs in treated LoVo and DLD‐1 cells. (D) Anti‐Ago2 RIP assay was executed in LoVo cells after transfection with miR‐576‐5p mimics and mimics NC, followed by qRT‐PCR to detect the expression of hsa_circ_0001666. (E) qRT‐PCR was used to identify the relative expression of miR‐576‐5p in CRC tissues and matched normal tissues (*n* = 70). (F) Pearson correlation analysis of hsa_circ_0001666 with miR‐576‐5p expression based on CRC tissues. (G) The locations of hsa_circ_0001666 (red) and miR‐576‐5p (green) in the cell were investigated using FISH. (magnification, 600×; scale bar, 100 μm). (H) The relative luciferase activities were detected in HEK‐293T cells after co‐transfection with hsa_circ_0001666‐WT or hsa_circ_0001666‐MUT and miR‐576‐5p mimics or mimics NC, respectively. Data were all represented as mean ± SD (*n* = 3); n.s indicated no significance, *p < .05, **p < .01

Considering that circRNAs serve as the sponge of microRNAs (miRNAs) primarily in the cytoplasm,^25^, ^26^ we then predicted the possible hsa_circ_0001666‐related miRNAs by two online databases (circBank and StarBase) and screened out seven miRNAs overlapped (Figure 4A). RNA pull‐down assay was subsequently performed with a biotinylated hsa_circ_0001666 probe or an NC probe. We filtered out four possible miRNAs which could be dragged down by hsa_circ_0001666 both in LoVo and DLD‐1 (Figure 4B) (*p*<.05). Then we compared the relative expression levels of four candidate miRNAs in LoVo‐NC/si‐circ‐1 and DLD‐1‐Vector/oe‐circ. Only miR‐576‐5p was both upregulated in LoVo cells and downregulated in DLD‐1 cells (Figure 4C) (*p*<.05). MiRNAs were previously thought to regulate target gene expression through binding to Ago2.^27^ Then, in LoVo cells, Ago2‐bound RNA transcripts were pulled down with the RIP assay. We demonstrated that anti‐Ago2 greatly pulled down the hsa_circ_0001666 compared with anti‐IgG. Furthermore, in cells transfected with miR‐576‐5p mimics, hsa_circ_0001666 was more abundant than those transfected with miR‐576‐5p mimics NC (Figure 4D) (*p*<.01). Moreover, the relative expression level of miR‐576‐5p in the previous 70 pairs of CRC tissues was upregulated in CRC and negatively related to hsa_circ_0001666 (Figure 4E,F) (*p*<.05). The subcellular localization of hsa_circ_0001666 as well as miR‐576‐5p was then observed by the FISH assay in LoVo and DLD‐1 cells. The findings discovered that the majority of miR‐576‐5p (green) and hsa_circ_0001666 (red) were placed in the cytoplasm (Figure 4G). Finally, a luciferase reporter assay was performed to determine whether hsa_circ_0001666 and miR‐576‐5p could bind directly. We predicted their binding sites in StarBase (chr6:170627329‐170627334[+]) and made mutations. Hsa_circ_0001666‐WT and hsa_circ_0001666‐MUT were co‐transfected with miR‐576‐5p mimics or mimics NC in the pmiR‐RB‐Report luciferase reporter vector. The luciferase activity of the reporter vector containing the hsa_circ_0001666‐WT sequence decreased significantly in the miR‐576‐5p mimics group, but there was no discernible change in that containing the hsa_circ_0001666‐MUT sequence (Figure 4H) (*p*<.01). These experiments explained hsa_circ_0001666 might bind directly to miR‐576‐5p for regulating the miR‐576‐5p expression in CRC cells.

### 3.5 Hsa_circ_0001666 suppresses CRC progression depending on miR‐576‐5p

Next, we examined miR‐576‐5p functions. First, we investigated the miR‐576‐5p expression in seven CRC cell lines and NCM460 (Figure S3A in the Supporting Information) (*p*<.05). MiR‐576‐5p was maximally expressed in DLD‐1 and minimally expressed in LoVo cells. Thus, we transfected miR‐576‐5p mimics or miR‐576‐5p mimics NC into LoVo cells and miR‐576‐5p inhibitor or miR‐576‐5p inhibitor NC into DLD‐1 cells. Both mimics and inhibitors were successfully constructed (Figure S3B,C in the Supporting Information) (*p*<.01).

Upregulation of miR‐576‐5p substantially increased the cells proliferation in CCK‐8 assays, while its downregulation had the opposite impact (Figure S4A in the Supporting Information) (*p* < .05). Moreover, colony formation assays revealed that miR‐576‐5p mimics significantly facilitated the cloning of LoVo cells, while the miR‐576‐5p inhibitor markedly inhibited it (Figure S4B in the Supporting Information) (*p*<.01). Similarly, EdU assays showed that miR‐576‐5p upregulation raised the percentages of EdU‐positive cells, while downregulation of miR‐576‐5p reduced it (Figure S4C in the Supporting Information) (*p*<.01). These results indicated that miR‐576‐5p facilitated CRC cell proliferation.

Then, the apoptosis assays suggested the apoptotic rate was reduced in LoVo cells transfected with miR‐576‐5p mimics, while increased in DLD‐1 cells transfected with miR‐576‐5p inhibitor (Figure S4D in the Supporting Information) (*p*<.05). This experiment suggested that miR‐576‐5p suppressed the CRC cells apoptosis.

Finally, wound healing and transwell assays revealed that upregulation of miR‐576‐5p greatly enhanced the migratory and invasive abilities of LoVo cells, while downregulation of miR‐576‐5p remarkably suppressed them in DLD‐1 cells (Figure S4E,F in the Supporting Information) (*p*<.01). These findings presented that miR‐576‐5p enhanced migration as well as invasion of CRC cells.

**FIGURE 5:**
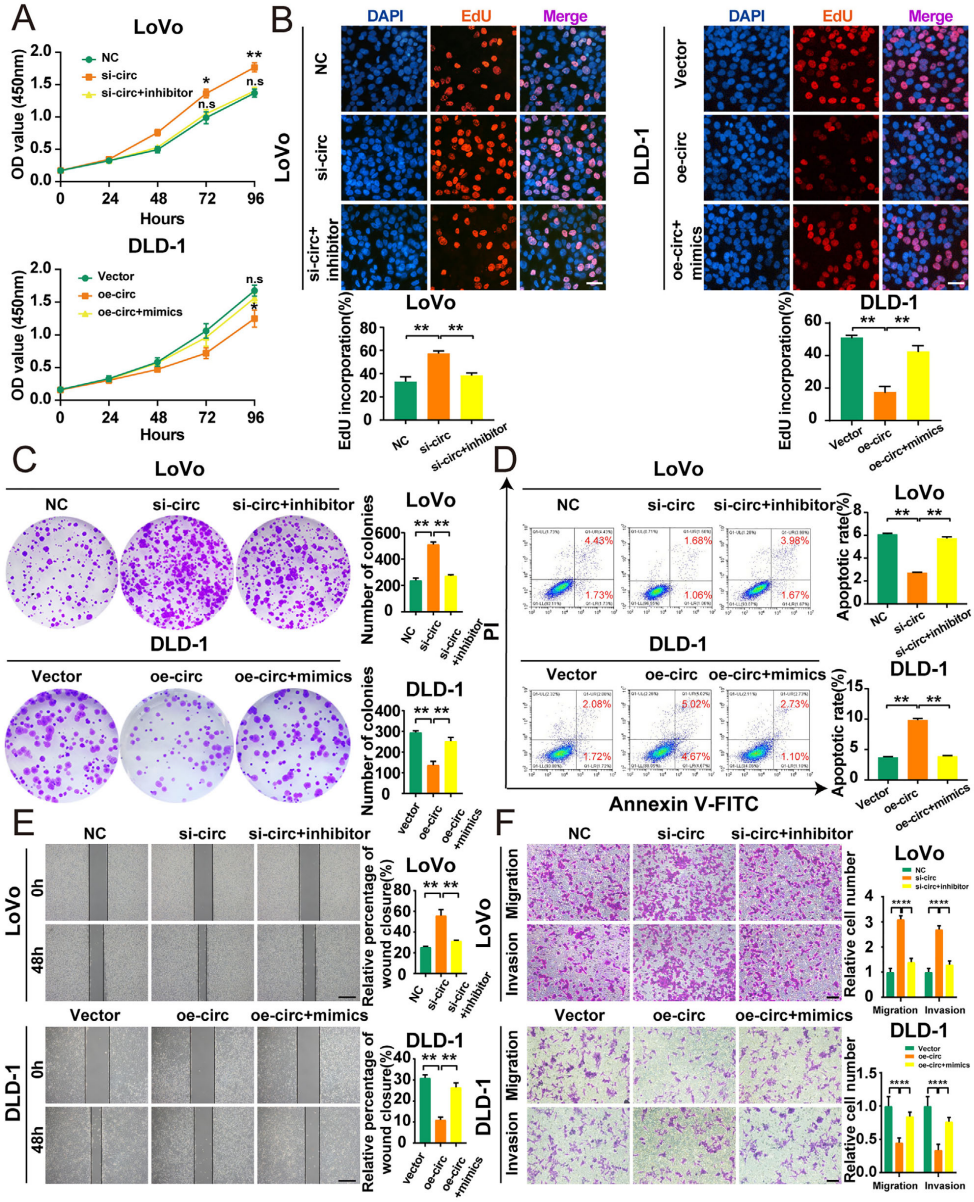
Hsa_circ_0001666 suppresses CRC progression partially depending on miR‐576‐5p. LoVo cells were transfected with NC, si‐circ, si‐circ+miR‐inhibitor, and DLD‐1 cells were transfected with Vector, oe‐circ, oe‐circ+miR‐mimics. (A–C) To test the proliferative ability, CCK‐8 assays, colony formation assays, and EdU assays were used. (magnification, 200×; scale bar, 100 μm). (D) The Annexin‐V FITC/PI staining was used to assess the apoptotic rates. (E, F) Wound healing assays and transwell assays were used to determine cell migratory and invasive capabilities. The scale bar in wound healing assays indicated 20 μm; the scale bar in transwell assays indicated 200 μm. Data were all represented as mean ± SD (*n* = 3). n.s indicated no significance, *p < .05, **p < .01

To clarify whether the function of hsa_circ_0001666 for CRC was dependent on miR‐576‐5p, si‐hsa_circ_0001666‐1 and miR‐576‐5p inhibitor were transfected into LoVo cell lines, and oe‐hsa_circ_0001666 and miR‐576‐5p mimic into DLD‐1 cell lines. Then we repeated the above‐mentioned functional experiments. CCK‐8 assay, EdU assay, as well as colony formation assay presented that inhibiting miR‐576‐5p partially alleviated the increase of cell viability caused by hsa_circ_0001666 downregulation (Figure 5A–C) (*p*<.05).

Inhibition of miR‐576‐5p could reverse si‐hsa_circ_0001666‐enhanced downregulation of the apoptotic rate in CRC cells, according to apoptosis assays (Figure 5D) (*p*<.01).

Similarly, wound healing and transwell assays showed the miR‐576‐5p inhibition could reverse the si‐hsa_circ_0001666‐induced upregulation of migration and invasion (Figure 5E,F) (*p*<.01).Opposite results were observed in DLD‐1 cells. These experiments illustrated miR‐576‐5p could regulate the hsa_circ_0001666 effect on CRC cells.

### 3.6 PCDH10 is a direct target of miR‐576‐5p in CRC

**FIGURE 6:**
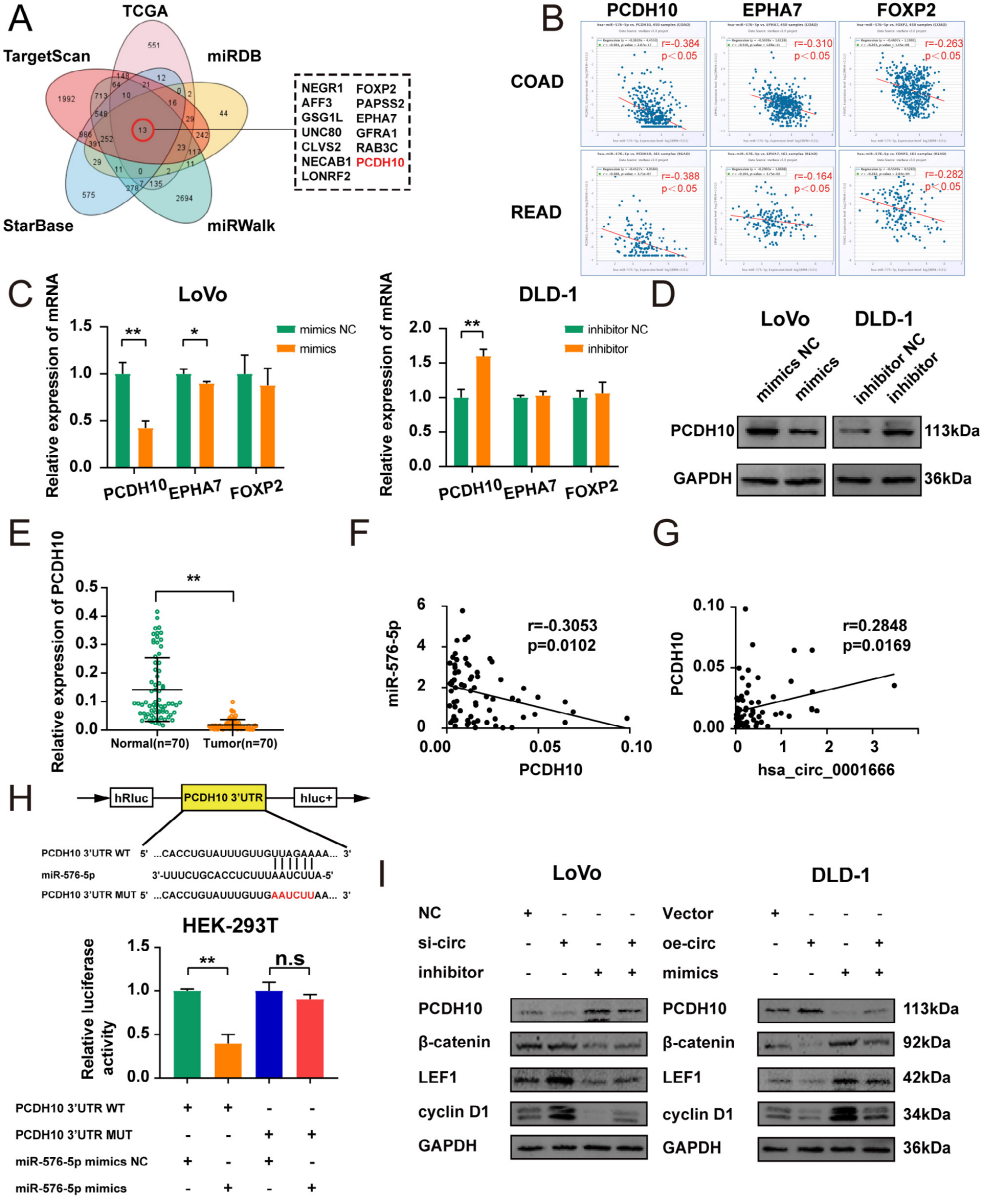
PCDH10 is a direct target of miR‐576‐5p in CRC. (A) Overlapped target genes of miR‐576‐5p predicted by TargetScan, StarBase, miRWalk, miRDB, TCGA. (B) Pearson correlation analysis of miR‐576‐5p with PCDH10/EPHA7/FOXP2 in StarBase. (C) Relative expression of three candidate mRNAs in treated LoVo and DLD‐1 cells by qPCR. (D) Relative expression of PCDH10 in treated LoVo and DLD‐1 cells by Western blot. (E) Relative expression of PCDH10 in CRC tissues and matched adjacent normal tissues (*n* = 70). (F, G) Pearson correlation analysis of miR‐576‐5p with PCDH10, and hsa_circ_0001666 with PCDH10 in CRC tissues. (H) After co‐transfection with PCDH10 3′UTR‐WT or PCDH10 3′UTR‐MUT and miR‐576‐5p mimics or mimics NC, relative luciferase activities were observed in HEK‐293T cells. (I) Several markers of the Wnt/β‐catenin signalling pathway were tested by Western blot in LoVo cells transfected with NC, si‐circ, miR‐inhibitor and si‐circ+miR‐inhibitor and DLD‐1 cells transfected with Vector, oe‐circ, miR‐mimics and oe‐circ+miR‐mimics. Data were all represented as mean ± SD (*n* = 3); n.s indicated no significance, *p < .05, **p < .01

Four online databases (TargetScan, StarBase, miRDB, miRWalk) were utilized for predicting the target genes that miR‐576‐5p could bind to in 3′UTR. The result was combined with the downregulated genes in CRC selected in TCGA databases, and a total of 13 overlapped genes were found from five databases (Figure 6A). Then the genes negatively related to miR‐576‐5p in both colon and rectum cancer were further filtered out by searching the StarBase Database. Three genes (PCDH10, EPHA7, FOXP2) were selected (Figure 6B). To validate this finding, the mRNA expressions of the three selected genes were analysed in LoVo and DLD‐1 cell lines transfected with miR‐576‐5p mimics as well as an inhibitor, respectively. Only PCDH10 was dysregulated in both DLD‐1 and LoVo cell lines (Figure 6C) (*p*<.05); meanwhile, overexpressing or inhibiting miR‐576‐5p could also reduce or enhance the expression of PCDH10 at the protein level (Figure 6D; Figure S5A in the Supporting Information) (*p*<.01). The expressions of hsa_circ_0001666, miR‐576‐5p and PCDH10 from 70 CRC tissues showed a negative correlation between miR‐576‐5p and PCDH10 and positive correlation between hsa_circ_0001666 and PCDH10 (Figure 6E–G) (*p*<.05). To explore whether miR‐576‐5p could directly interact with PCDH10, we developed 3′‐UTR sensors as well as co‐transfected miR‐576‐5p mimics into HEK‐293T cells. Overexpression of miR‐576‐5p resulted in decreased luciferase activity by the PCDH10 3′‐UTR. However, when we mutated the sequence of miR‐576‐5p binding to PCDH10 3′‐UTR, much higher luciferase activity was found (Figure 6H) (*p*<.01). Interestingly, the binding site between PCDH10 mRNA as well as miR‐576‐5p was the same as that between miR‐576‐5p along with hsa_circ_0001666. These findings also recommended that in CRC cells PCDH10 was co‐expressed with hsa_circ_0001666 and miR‐576‐5p and could bind directly to miR‐576‐5p.

Several studies indicated that PCDH10 could inhibit the canonical Wnt/β‐catenin signalling pathway, a well‐known cancer‐promoting pathway. Therefore, we detected the protein level of PCDH10, as well as β‐catenin, LEF1, and cyclin D1(the markers in the Wnt/β‐catenin signalling pathway). Western blot demonstrated that the Wnt/β‐catenin signalling pathway was suppressed by hsa_circ_0001666 overexpression, while miR‐576‐5p mimics could reverse this event (Figure 6I; Figure S5B,C in the Supporting Information) (*p*<.05). These results revealed that hsa_circ_0001666 could suppress the Wnt/β‐catenin signalling pathway via inducing PCDH10 expression.

### 3.7 PCDH10 regulates the function of hsa_circ_0001666 on the proliferation, apoptosis and invasion of CRC cells

**FIGURE 7:**
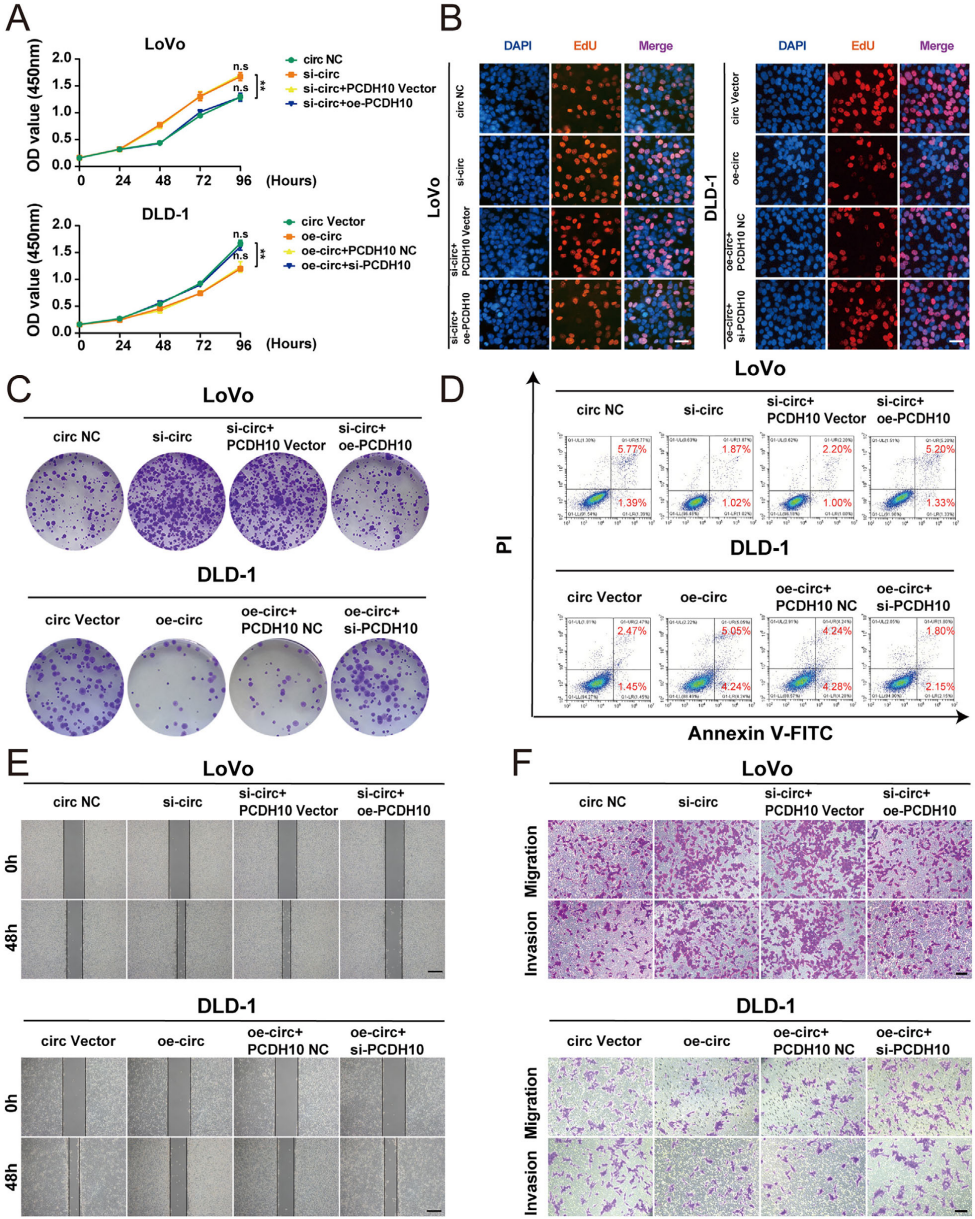
PCDH10 regulates the function of hsa_circ_0001666 on the proliferation, apoptosis and invasion of CRC cells. LoVo cells were transfected with circ NC, si‐circ, si‐circ+PCDH10 Vector, si‐circ+oe‐PCDH10, and DLD‐1 cells were transfected with circ Vector, oe‐circ, oe‐circ+PCDH10 NC, oe‐circ+si‐PCDH10. (A–C) To test the proliferative ability, CCK‐8 assays, EdU assays and colony formation assays were used (magnification, 200×; scale bar, 100 μm). (D) The Annexin‐V FITC/PI staining was used to assess the apoptotic rates. (E,F) Wound healing assays and transwell assays were performed to explore the migratory and invasive capabilities. The scale bar in wound healing assays indicated 20 μm; the scale bar in transwell assays indicated 200 μm

To further investigate whether PCDH10 could influence the hsa_circ_0001666 suppression in CRC progression. We expressed the oe‐PCDH10 vector in LoVo cells transfected with si‐hsa_circ_0001666‐1, and si‐PCDH10 in DLD‐1 cells transfected with oe‐hsa_circ_0001666. CCK‐8 assay, EdU assay and colony formation assay showed downregulating hsa_circ_0001666 improved cell vitality and growth, but overexpressed PCDH10 could inhibit the proliferative capacity of LoVo cells, and the opposite results were found in DLD‐1 cells (Figure 7A–C; Figure S6A–D in the Supporting Information) (*p*<.01). Flow cytometry for apoptosis showed that knocking down hsa_circ_0001666 suppressed cell apoptosis, but this suppression was blocked by PCDH10 overexpression. Opposite results were observed in DLD‐1 cells (Figure 7D; Figure S6E,F in the Supporting Information) (*p*<.01). Moreover, wound healing and transwell assays demonstrated upregulating or downregulating PCDH10 could recover the migrative and invasive abilities in LoVo cells transfected with si‐hsa_circ_0001666‐1 or DLD‐1 cells transfected with oe‐hsa_circ_0001666 (Figure 7E,F; Figure S6G‐J in the Supporting Information) (*p*<.01). All these findings indicated that PCDH10 was a tumour suppressor gene through regulating the function of hsa_circ_0001666 on the proliferation, apoptosis as well as invasion of CRC cells.

### 3.8 Hsa_circ_0001666 suppresses EMT and cell stemness by miR‐576‐5p/PCDH10 axis in vitro

**FIGURE 8:**
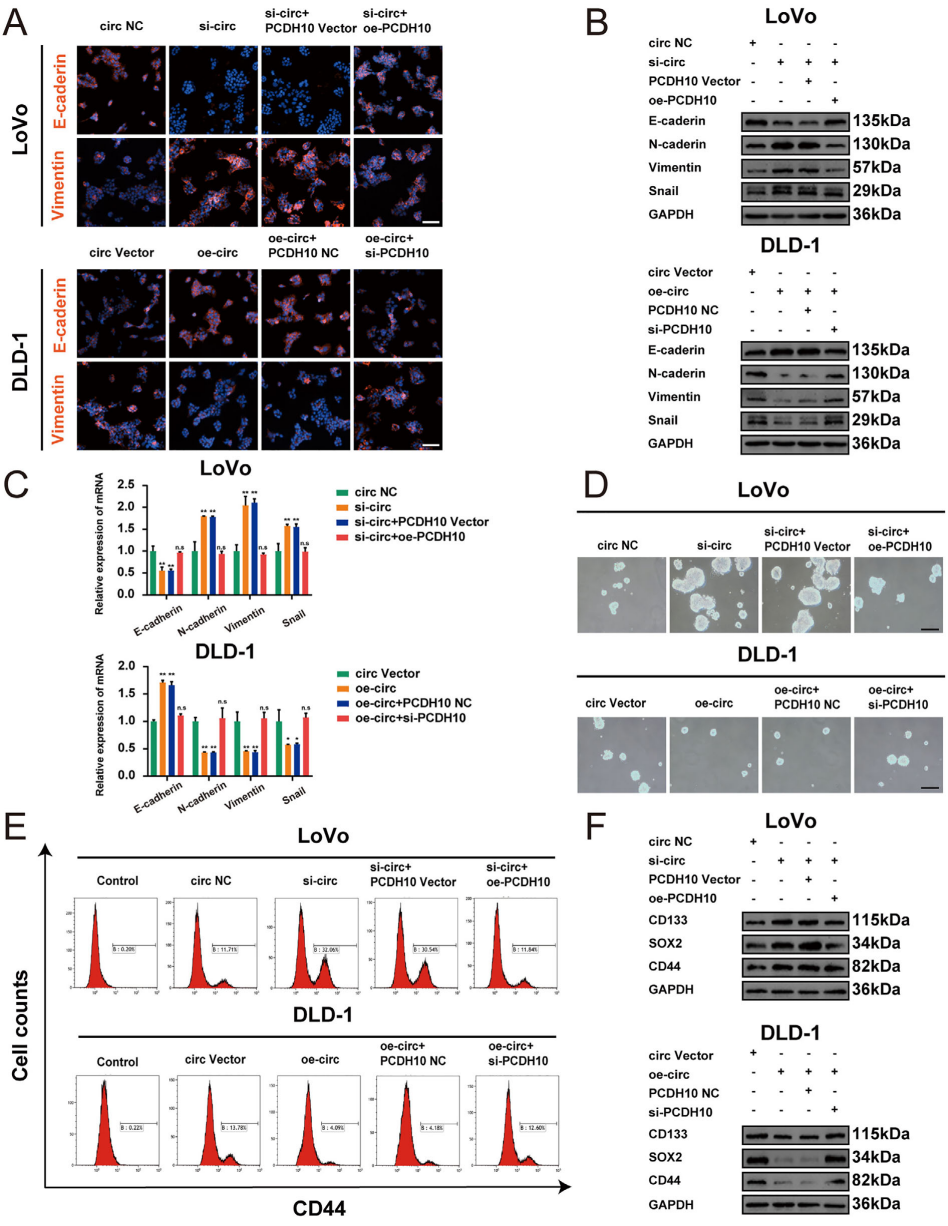
Hsa_circ_0001666 suppresses EMT and cell stemness by miR‐576‐5p/PCDH10 axis in vitro. LoVo cells were transfected with circ NC, si‐circ, si‐circ+PCDH10 Vector, si‐circ+oe‐PCDH10, and DLD‐1 cells were transfected with circ Vector, oe‐circ, oe‐circ+PCDH10 NC, oe‐circ+si‐PCDH10. (A) The expression of E‐cadherin and Vimentin were detected using IF; scale bars, 50 μm. (B,C) The expression of EMT marker genes was detected by Western blot and qRT‐PCR. (D) Typical images from the sphere formation assay. (E) The number of cells with the CD44+ phenotype. (F) The expression of stemness marker genes was detected by Western blot. Data were represented as mean ± SD (*n* = 3); n.s indicated no significance, **p < .01

PCDH10 has been reported to be a negative regulator to suppress EMT and cell stemness.^22^ Given these results, we wondered whether hsa_circ_0001666 could inhibit EMT and stemness by miR‐576‐5p/PCDH10 axis. We co‐transferred si‐hsa_circ_0001666‐1/oe‐PCDH10 or si‐hsa_circ_0001666‐1/ PCDH10 Vector and oe‐hsa_circ_0001666/si‐PCDH10 or oe‐hsa_circ_0001666/ PCDH10 NC into LoVo and DLD‐1 cell lines, respectively. Immunofluorescence (IF) was used to detect E‐cadherin (a marker of epithelial cells) and Vimentin (a marker of mesenchymal cells). We demonstrated that knocking down of hsa_circ_0001666 could suppress the E‐cadherin expression, however, raise Vimentin expression, while overexpressing PCDH10 could partly reverse the EMT progress (Figure 8A; Figure S7A in the Supporting Information) (*p*<.001). The EMT markers were also detected by qRT‐PCR and Western blot, whereas findings were consistent with IF (Figure 8B, C; Figure S7B in the Supporting Information) (*p*<.01). Then we performed a soft agar assay, and the results showed the number of spheres was significantly raised after hsa_circ_0001666 downregulation and decreased when PCDH10 was overexpressed (Figure 8D; Figure S7C in the Supporting Information) (*p*<.01). Cancer cells with CD44+ on their membranes have stem cell‐like characteristics as well as the ability to self‐renew. The CD44+ level were increased in the hsa_circ_0001666 inhibition group; meanwhile, upregulating PCDH10 could lower the level of CD44+. Opposite results were found in DLD‐1 cells (Figure 8E; Figure S7D in the Supporting Information) (*p*<.01). Furthermore, the protein levels of CD133, SOX2 and CD44 (markers of stemness) were increased when hsa_circ_0001666 was knocked down, and PCDH10 upregulation could decrease the expression level of these markers. Opposite findings were found in DLD‐1 cell lines (Figure 8F; Figure S7E in the Supporting Information) (*p*<.05). These experiments showed EMT and cell stemness were inhibited by hsa_circ_0001666 and reversed by PCDH10.

**FIGURE 9:**
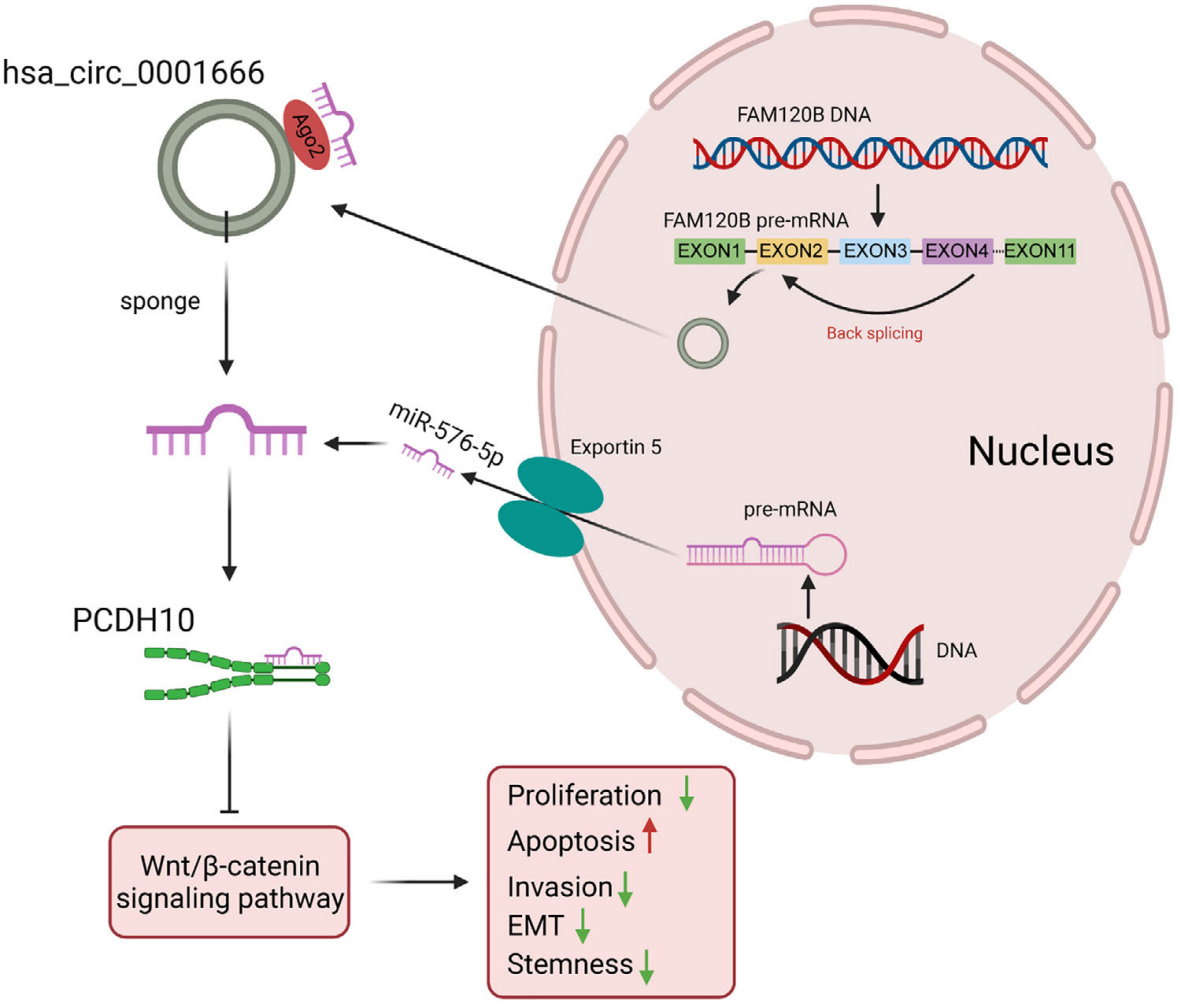
Molecular mechanism of hsa_circ_0001666 in the progression of CRC

In conclusion, these experiments revealed that hsa_circ_0001666 suppressed CRC development via boosting PCDH10 expression (Figure 9).

## 4 DISCUSSION

CircRNA is a non‐coding RNA that has gained a lot of attention in recent years. Current studies have shown the functions of circRNAs in a variety of biological processes.^28^, ^29^, ^30^ Numerous circRNAs are expressed differentially in CRC as well as matched normal tissues.^31^ In this research, we observed that hsa_circ_0001666 was downregulated dramatically in CRC cells and tissues. This is the first research to explore the function, expression as well as clinical significance of hsa_circ_0001666 in CRC to our knowledge. Moreover, this is also the first to discover a connection among hsa_circ_0001666, miR‐576‐5p and PCDH10.

First, through circRNA microarray analysis based on the GEO database, we discovered hsa_circ_0001666 that was significantly downregulated in CRC whereas negatively connected with the TNM stage along with overall survival of CRC patients. Second, hsa_circ_0001666 functioned as a tumour suppressor through inhibiting CRC cell proliferation, metastasis, EMT progression and stemness as well as inducing CRC cell apoptosis. Third, miR‐576‐5p was upregulated in CRC cells and tissues as well as negatively associated with expression of hsa_circ_0001666. Fourth, mechanistic experiments indicated that hsa_circ_0001666 regulated the CRC activities through sponging miR‐576‐5p and reducing its inhibitory effect on the target gene PCDH10. These findings highlighted the potentiality of hsa_circ_0001666 for CRC therapy.

Subcellular localization of circRNAs determines their functions in cells. FISH assays showed hsa_circ_0001666 was mainly situated in the cytoplasm, where circRNAs function as miRNA sponge, interact with RBP or encode the polypeptide.^11^, ^12^, ^13^ Since most circRNAs contain miRNA response elements (MREs), it has been proposed that circRNAs can function like a ‘miRNA sponge' and form a ‘circRNA‐miRNA‐mRNA’ axis.^32^, ^33^, ^34^ For example, CDR1a harbours 63 conserved binding sites for miR‐7 and antagonizes miRNA.^3^ In the present research, we utilized bioinformatics tools for teasing out seven possible miRNAs with multiple binding sites for hsa_circ_0001666. FISH, RIP, RNA pull‐down as well as luciferase reporter assays have been widely utilized for determining the interactions between circRNAs and miRNAs.^35^ MiR‐576‐5p directly bound to hsa_circ_0001666 in CRC progression and metastasis. Furthermore, we discovered that upregulating miR‐576‐5p expression inhibited PCDH10 activation in CRC cells, implying that miR‐576‐5p is a negative regulator of PCDH10. Therefore, we infer that hsa_circ_0001666 acts as a miR‐576‐5p sponge and a promising therapeutic target for CRC.

PCDH10 is repressed by promoter hypermethylation in multiple cancers and functions as a tumour suppressor gene.^36^, ^37^, ^38^, ^39^ Accumulating studies showed PCDH10 was involved in cell growth, invasion, metastasis and apoptosis through Wnt/β‐catenin and PI3K/AKT signalling pathway.^21^, ^23^, ^40^ A recent research revealed that PCDH10 directly participated in the negative regulation of the EGFR/AKT/β‐catenin signalling pathway, therefore inhibiting the EMT and stemness of CRC cells.^22^ However, the upstream regulation of PCDH10 remains indefinite. In our study, we determined that hsa_circ_0001666 suppressed the proliferation, invasion, metastasis and induced apoptosis, as well as inhibited the EMT and stemness through targeting PCDH10 in CRC cells. Interestingly, the hsa_circ_0001666/miR‐576‐5p/PCDH10 axis also interfered with the Wnt/β‐catenin signalling pathway.

The interpretation of our findings, however, has several limitations. First, our study revealed hsa_circ_0001666 could play a tumour‐suppressive role in vitro and in vivo; thus, further investigation of hsa_circ_0001666 in the human body is also needed. Second, other miRNAs which have not been predicted by bioinformatics analysis may also bind to hsa_circ_0001666 to function in CRC. Third, hsa_circ_0001666 may employ other mechanisms to regulate the development of CRC. As a result, additional research is needed to gain a better understanding of hsa_circ_0001666 in CRC.

## 5 CONCLUSIONS

Hsa_circ_0001666 expression is significantly reduced in cell lines and CRC tissues. By sponging miR‐576‐5p and reducing its suppression on PCDH10, hsa_circ_0001666 inhibits the progression of CRC. For CRC patients, hsa_circ_0001666 may be expected to be applied to replacement therapy.

## CONFLICT OF INTEREST

The authors declare no competing interests.

## Supporting information

Figure S1. Expressions of candidate circRNAs. (A–C) Relative expression of hsa_circ_0043278, hsa_circ_0000977 and hsa_circ_0006220 in CRC tissues and matched adjacent normal tissues (n = 70). Data were showed as mean ± SD; n.s indicated no significance, *P < 0.05.

Figure S2. Construction of cell lines knocking down and overexpressing hsa_circ_0001666. (A) Relative expression of hsa_circ_0001666 in normal colon epithelial cell lines and seven CRC cell lines were detected by qRT‐PCR. (B,C) The relative expression of hsa_circ_0001666 and liner mRNA in LoVo and DLD‐1 cell lines transfected with si‐hsa_circ_0001666/NC and oe‐hsa_circ_0001666/Vector. Data were showed as mean ± SD (n = 3), **P < 0.01, ***P < 0.001.

Figure S3. Construction of cell lines knocking down and overexpressing miR‐576‐5p. (A) Relative expression of miR‐576‐5p in normal colon epithelial cell line and seven CRC cell lines was detected by qRT‐PCR. (B,C) The relative expression of miR‐576‐5p in LoVo and DLD‐1 cell lines transfected with mimics/mimics NC and inhibitor/inhibitor NC. Data were showed as mean ± SD (n = 3), **P < 0.01, *P < 0.05.

Figure S4. miR‐576‐5p promotes the proliferation, invasion and inhibits the apoptosis of CRC cells in vitro. LoVo cells were transfected with mimics, mimics NC and DLD‐1 cells were transfected with inhibitor, inhibitor NC. (A‐C) To assess the proliferative ability, CCK‐8 assays, colony‐forming assays and EdU assays were used. (magnification, 200×; scale bar, 100 μm). (D) The Annexin‐V FITC/PI staining was used to assess apoptotic rates. (E, F) Wound healing assays and transwell assays were used to determine the migratory and invasive capabilities. The scale bar in wound healing assays indicated 20 μm; the scale bar in transwell assays indicated 200 μm. Data were all showed as mean ± SD (n = 3). *P < 0.05, **P < 0.01.

Figure S5. PCDH10 is a direct target of miR‐576‐5p in CRC. (A) The relative expression of PCDH10 in LoVo cells transfected with mimics mimics NC and DLD‐1 cells transfected with inhibitor, inhibitor NC by Western blot. (B,C) The relative expression of PCDH10, β‐catenin, LEF1, cyclin D1 in LoVo cells transfected with NC/si‐circ/miR‐inhibitor/si‐circ+inhibitor and DLD‐1 cells transfected with circ Vector/oe‐circ/miR‐mimics/oe‐circ+mimics by Western blot.

Figure S6. PCDH10 regulates the function of hsa_circ_0001666 on the proliferation, apoptosis and invasion of CRC cells. LoVo cells were transfected with circ NC/si‐circ/si‐circ+PCDH10 Vector/si‐circ+oe‐PCDH10 and DLD‐1 cells were transfected with circ Vector/oe‐circ/oe‐circ+PCDH10 NC/oe‐circ+si‐PCDH10. (A,B) The histogram of EdU incorporation. (C,D) The histogram of colony numbers. (E.F) The histogram of apoptotic rate. (G,H) The histogram of wound closure percentage. (I,J) The histogram of relative cell number in a transwell assay. Data were all showed as mean ± SD (n = 3). n.s indicated no significance, **P < 0.01, ***P < 0.001.

Figure S7. Hsa_circ_0001666 suppresses EMT and cell stemness by miR‐576‐5p/PCDH10 axis in vitro. LoVo cells were transfected with circ NC/si‐circ/si‐circ+PCDH10 Vector/si‐circ+oe‐PCDH10 and DLD‐1 cells were transfected with circ Vector/oe‐circ/oe‐circ+PCDH10 NC/oe‐circ+si‐PCDH10. (A) The relative fluorescence intensity of E‐cadherin and Vimentin tested by IF. (B)The relative expression of EMT markers tested by Western blot. (C) The number of spheres. (D) The number of cells with the CD44+. (E) The relative expression of stemness marker genes tested by Western blot.

Table S1. Primers used in the study

## Data Availability

The data that support the findings of this study are openly available in Gene Expression Omnibus (GEO) at https://doi.org/10.1038/s41388-019-0857-8, reference number GSE126094.
