# Supplementary figures and images for: Hsa_circ_0001666 suppresses the progression of colorectal cancer through the miR‐576‐5p/PCDH10 axis
Source: Clin Transl Med. 2021 Nov 4;11(11):e565. doi: 10.1002/ctm2.565 (PMC8567033; doi:10.1002/ctm2.565)

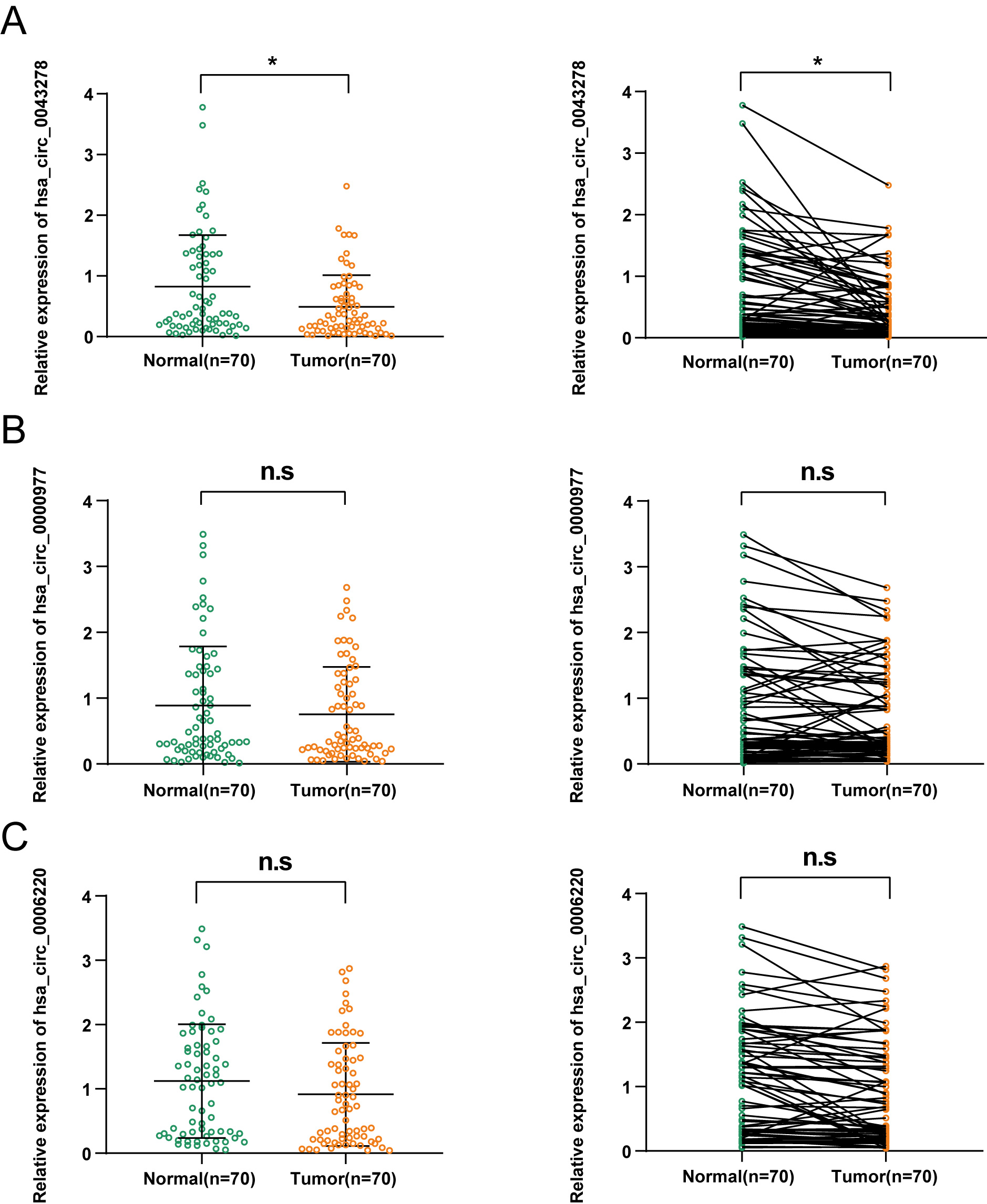

Supplement: Supplementary file 1 — Figure S1. Expressions of candidate circRNAs. (A–C) Relative expression of hsa_circ_0043278, hsa_circ_0000977 and hsa_circ_0006220 in CRC tissues and matched adjacent normal tissues (n = 70). Data were showed as mean ± SD; n.s indicated no significance, *P < 0.05. [file CTM2-11-e565-s007.jpg]

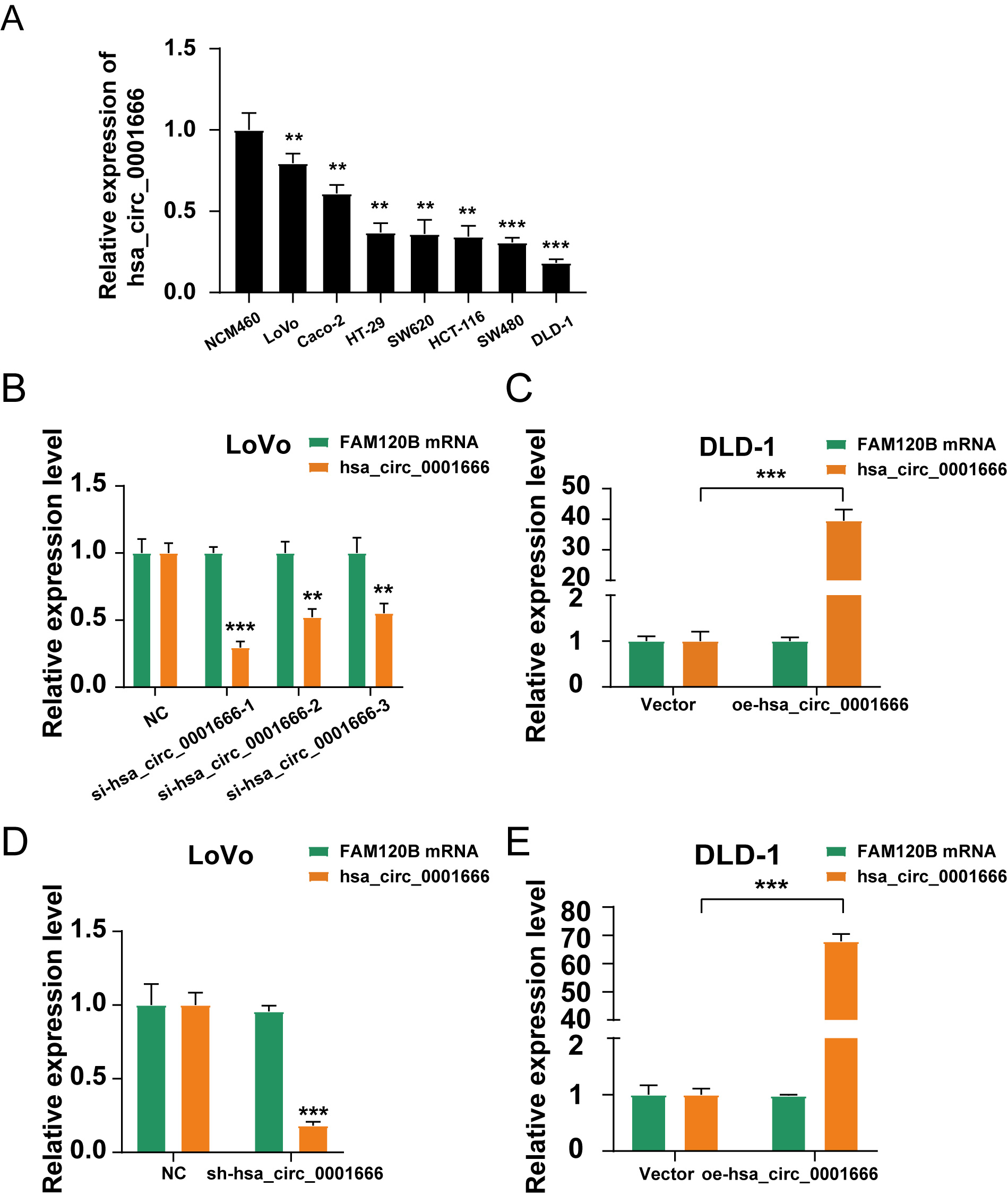

Supplement: Supplementary file 2 — Figure S2. Construction of cell lines knocking down and overexpressing hsa_circ_0001666. (A) Relative expression of hsa_circ_0001666 in normal colon epithelial cell lines and seven CRC cell lines were detected by qRT‐PCR. (B,C) The relative expression of hsa_circ_0001666 and liner mRNA in LoVo and DLD‐1 cell lines transfected with si‐hsa_circ_0001666/NC and oe‐hsa_circ_0001666/Vector. Data were showed as mean ± SD (n = 3), **P < 0.01, ***P < 0.001. [file CTM2-11-e565-s001.jpg]

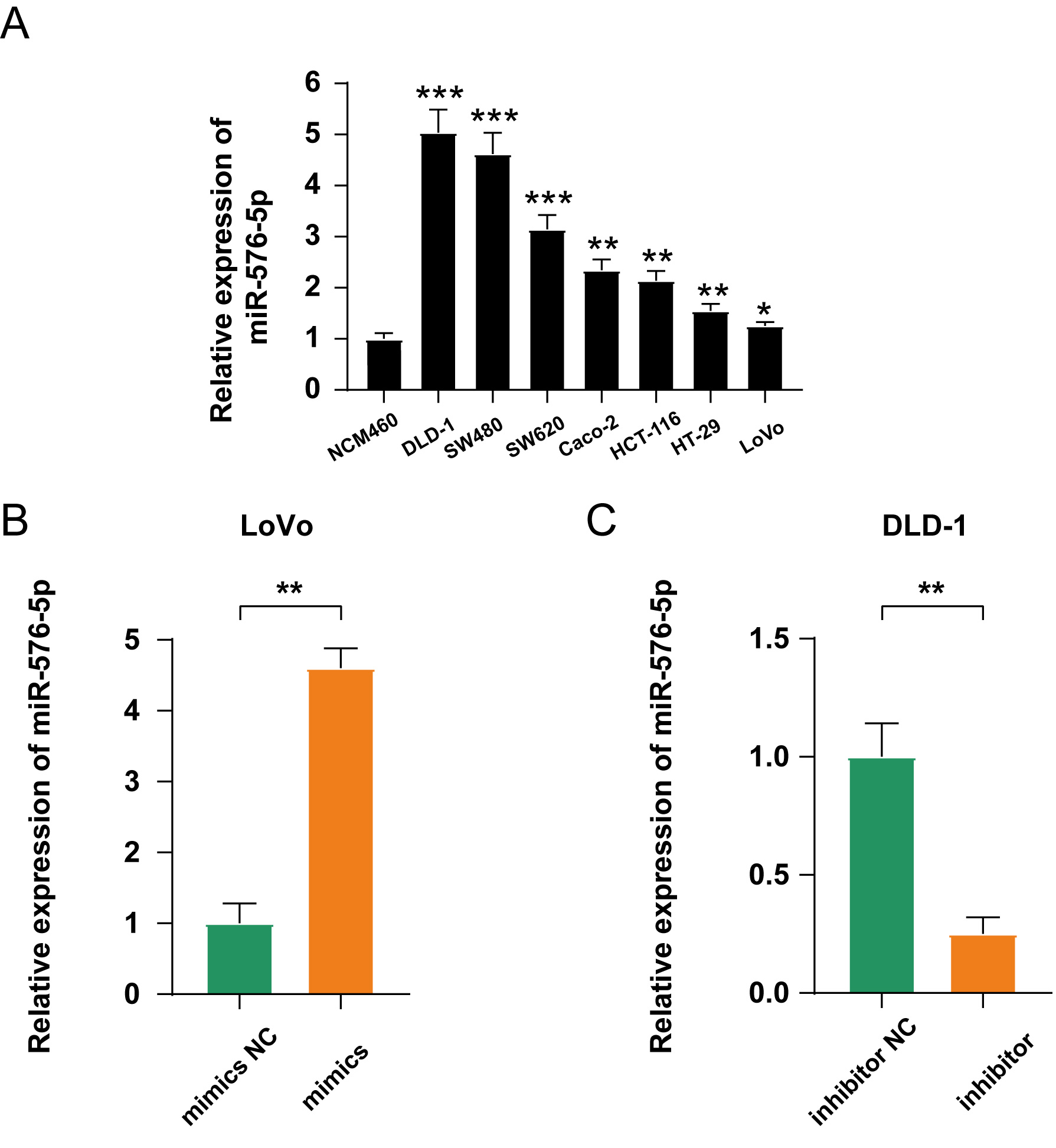

Supplement: Supplementary file 3 — Figure S3. Construction of cell lines knocking down and overexpressing miR‐576‐5p. (A) Relative expression of miR‐576‐5p in normal colon epithelial cell line and seven CRC cell lines was detected by qRT‐PCR. (B,C) The relative expression of miR‐576‐5p in LoVo and DLD‐1 cell lines transfected with mimics/mimics NC and inhibitor/inhibitor NC. Data were showed as mean ± SD (n = 3), **P < 0.01, *P < 0.05. [file CTM2-11-e565-s003.jpg]

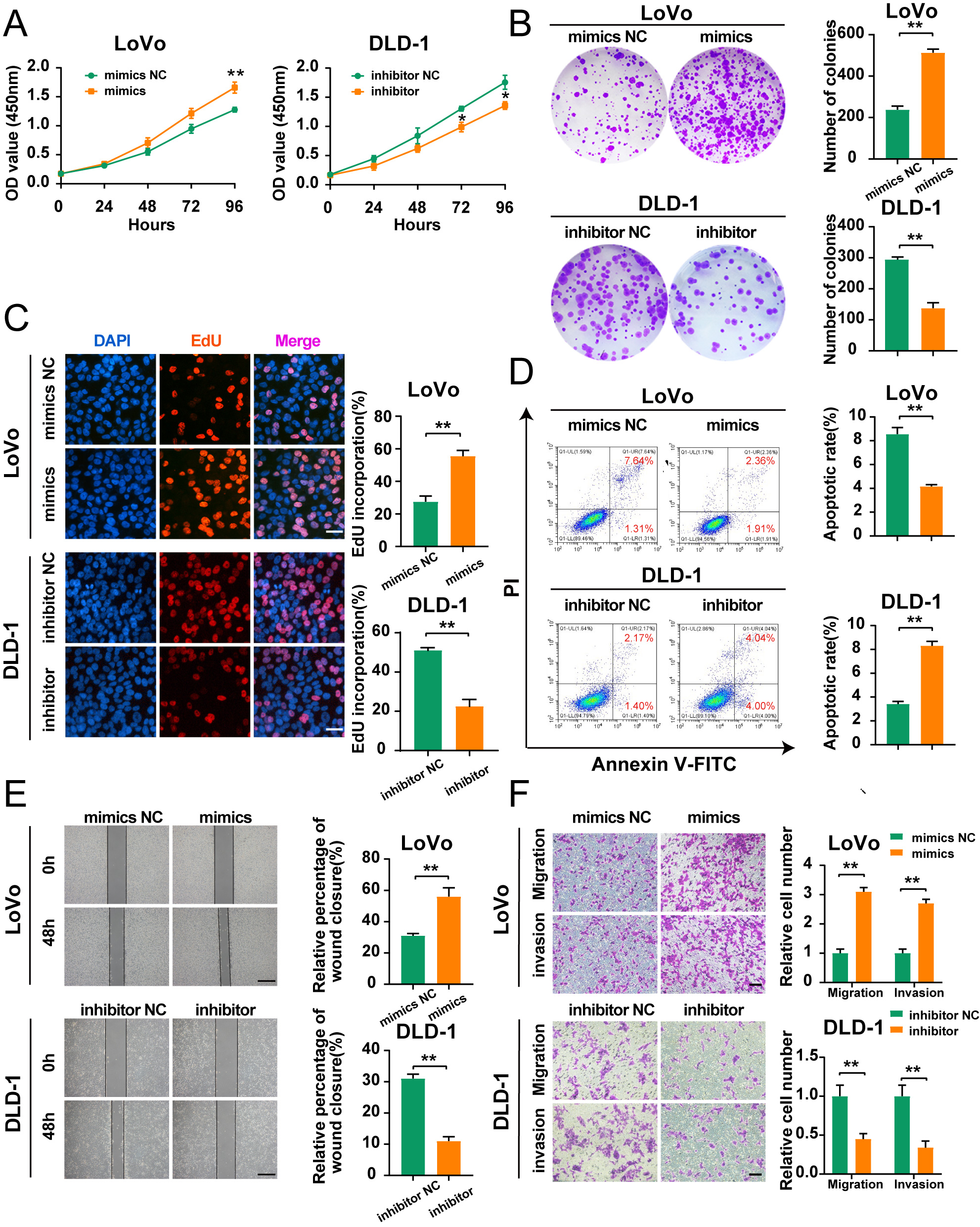

Supplement: Supplementary file 4 — Figure S4. miR‐576‐5p promotes the proliferation, invasion and inhibits the apoptosis of CRC cells in vitro. LoVo cells were transfected with mimics, mimics NC and DLD‐1 cells were transfected with inhibitor, inhibitor NC. (A‐C) To assess the proliferative ability, CCK‐8 assays, colony‐forming assays and EdU assays were used. (magnification, 200×; scale bar, 100 μm). (D) The Annexin‐V FITC/PI staining was used to assess apoptotic rates. (E, F) Wound healing assays and transwell assays were used to determine the migratory and invasive capabilities. The scale bar in wound healing assays indicated 20 μm; the scale bar in transwell assays indicated 200 μm. Data were all showed as mean ± SD (n = 3). *P < 0.05, **P < 0.01. [file CTM2-11-e565-s005.jpg]

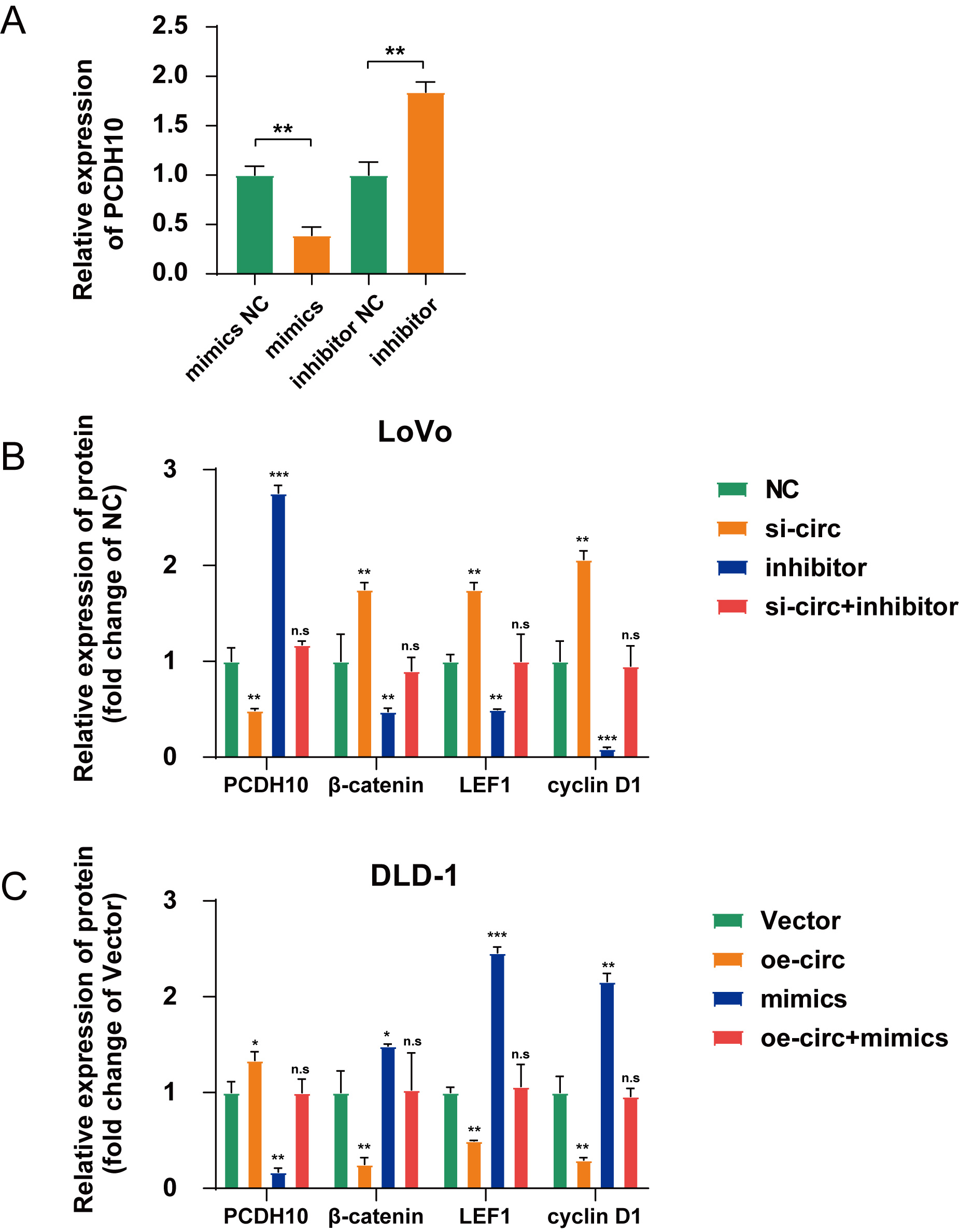

Supplement: Supplementary file 5 — Figure S5. PCDH10 is a direct target of miR‐576‐5p in CRC. (A) The relative expression of PCDH10 in LoVo cells transfected with mimics mimics NC and DLD‐1 cells transfected with inhibitor, inhibitor NC by Western blot. (B,C) The relative expression of PCDH10, β‐catenin, LEF1, cyclin D1 in LoVo cells transfected with NC/si‐circ/miR‐inhibitor/si‐circ+inhibitor and DLD‐1 cells transfected with circ Vector/oe‐circ/miR‐mimics/oe‐circ+mimics by Western blot. [file CTM2-11-e565-s008.jpg]

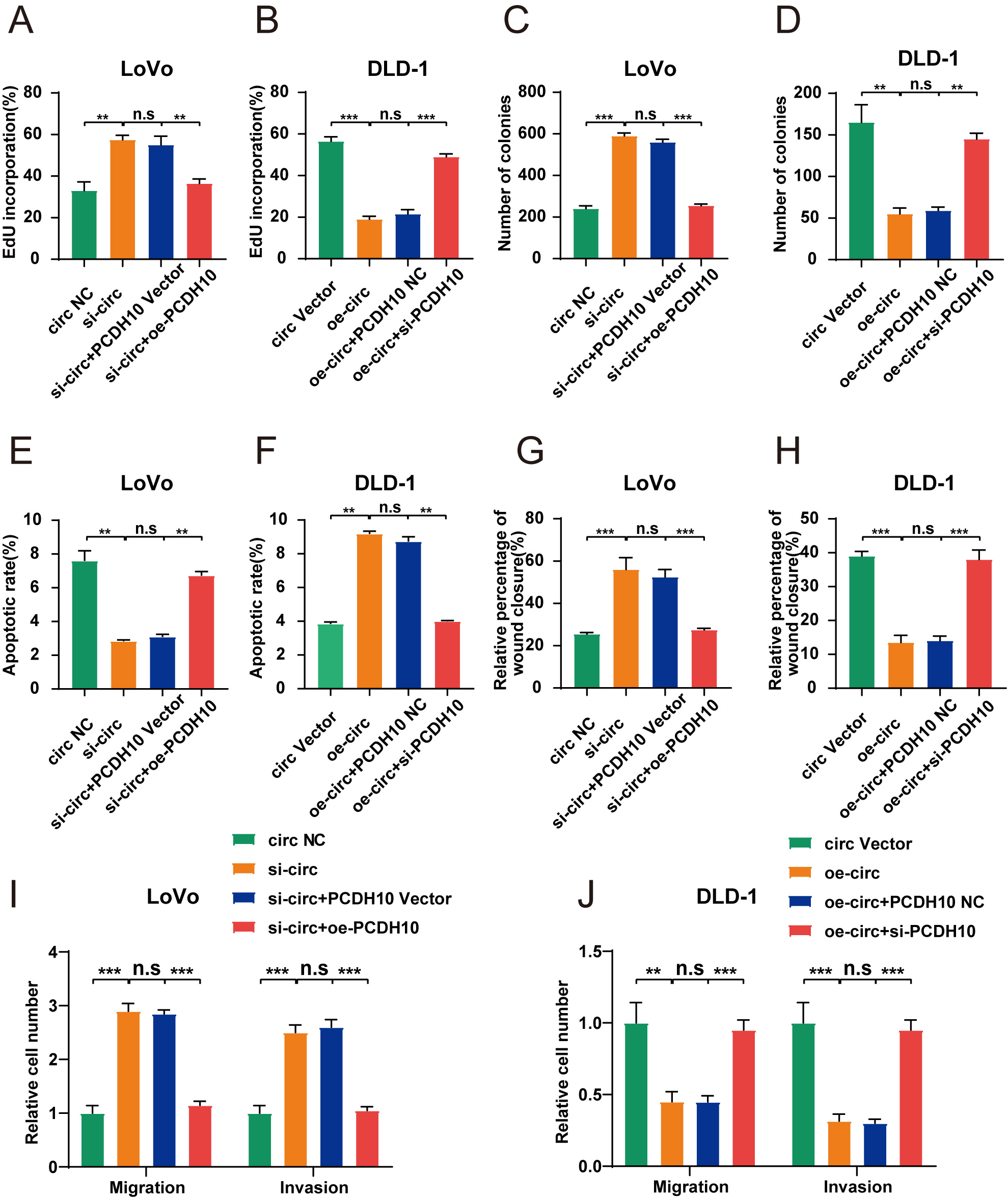

Supplement: Supplementary file 6 — Figure S6. PCDH10 regulates the function of hsa_circ_0001666 on the proliferation, apoptosis and invasion of CRC cells. LoVo cells were transfected with circ NC/si‐circ/si‐circ+PCDH10 Vector/si‐circ+oe‐PCDH10 and DLD‐1 cells were transfected with circ Vector/oe‐circ/oe‐circ+PCDH10 NC/oe‐circ+si‐PCDH10. (A,B) The histogram of EdU incorporation. (C,D) The histogram of colony numbers. (E.F) The histogram of apoptotic rate. (G,H) The histogram of wound closure percentage. (I,J) The histogram of relative cell number in a transwell assay. Data were all showed as mean ± SD (n = 3). n.s indicated no significance, **P < 0.01, ***P < 0.001. [file CTM2-11-e565-s002.jpg]

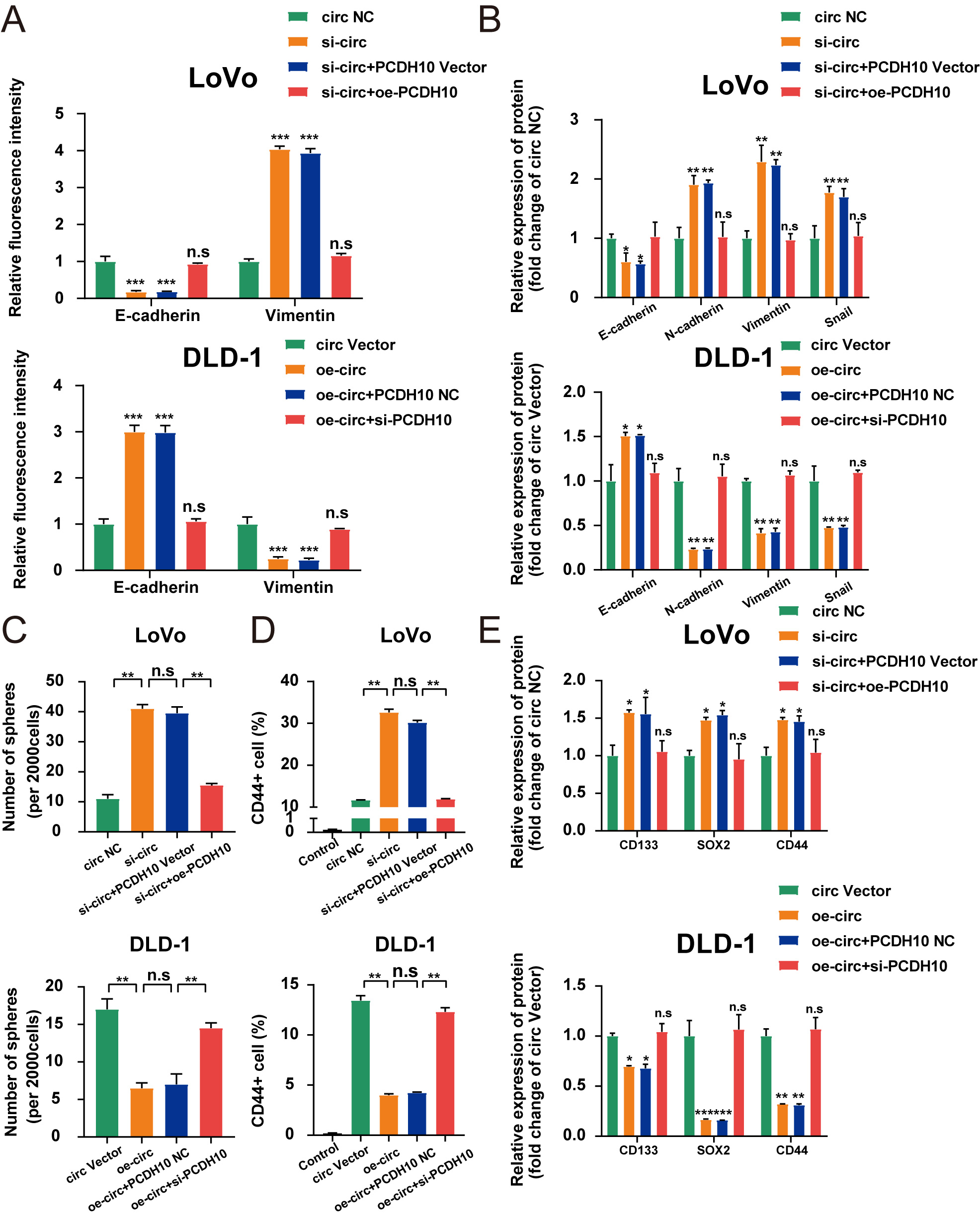

Supplement: Supplementary file 7 — Figure S7. Hsa_circ_0001666 suppresses EMT and cell stemness by miR‐576‐5p/PCDH10 axis in vitro. LoVo cells were transfected with circ NC/si‐circ/si‐circ+PCDH10 Vector/si‐circ+oe‐PCDH10 and DLD‐1 cells were transfected with circ Vector/oe‐circ/oe‐circ+PCDH10 NC/oe‐circ+si‐PCDH10. (A) The relative fluorescence intensity of E‐cadherin and Vimentin tested by IF. (B)The relative expression of EMT markers tested by Western blot. (C) The number of spheres. (D) The number of cells with the CD44+. (E) The relative expression of stemness marker genes tested by Western blot. [file CTM2-11-e565-s004.jpg]
