# Supplementary material for: Hsa_circ_0001666 suppresses the progression of colorectal cancer through the miR‐576‐5p/PCDH10 axis
Source: Clin Transl Med. 2021 Nov 4;11(11):e565. doi: 10.1002/ctm2.565 (PMC8567033; doi:10.1002/ctm2.565)
Supplement: Supplementary file 8 — Table S1. Primers used in the study [file CTM2-11-e565-s006.docx]

Table S1 Primers used in the study

| Primer | Sequence (5’-3’) |
| --- | --- |
| hsa_circ_0001666 F | ATGCCATGTGTTGTCTCAGATATTG |
| hsa_circ_0001666 R | TTCAGTGTCACCATGCCATCAA |
| hsa_circ_0043278 F | CTGTGATGGACTGTGGCTTTGGAA |
| hsa_circ_0043278 R | CTTGTTTCAGGTTCAGCAGGGTAGAT |
| hsa_circ_0000977 F | GGACGATACCTGAATCCTCTACAA |
| hsa_circ_0000977 R | CGGAGTTCTTATTCCACATCTTGAC |
| hsa_circ_0006220 F | AAGACCAAGGAGGAGTGTGAGAAG |
| hsa_circ_0006220 R | AGTGAAATGGAATGGCTGTGTCAG |
| FAM120B F | GAAGGAGAACAGACGCATCACT |
| FAM120B R | CCTGACTGGAACGGCTATACC |
| PCDH10 F | GGCTCGGTGTCCTTCATCTTCC |
| PCDH10 R | AGCAGCAGAGGCAGCAATCG |
| EPHA7 F | CTGTTATTATCATTGCTGTGGTTGCTGTAG |
| EPHA7 R | GCTCTTCATCGCCTTCTTGGTCAG |
| FOXP2 F | CAGCAGCAGCAGCAGCAACA |
| FOXP2 R | GGCAGCGATTGGACAGGAAGTG |
| GAPDH F | GAGAAGTATGACAACAGCCTCAAGAT |
| GAPDH R | ACGCCTGCTTCACCACCTT |
| miR-330-5p RT primer | GTCGTATCCAGTGCAGGGTCCGAGGTATTCGCACTGGATACGACGCCTAA |
| miR-330-5p F | GCGTCTCTGGGCCTGTGTC |
| miR-330-5p R | AGTGCAGGGTCCGAGGTATT |
| miR-548I RT primer | GTCGTATCCAGTGCAGGGTCCGAGGTATTCGCACTGGATACGACGGCAAA |
| miR-548I F | CGCGAAAAGTAATTGCGGAT |
| miR-548I R | AGTGCAGGGTCCGAGGTATT |
| miR-576-5p RT primer | GTCGTATCCAGTGCAGGGTCCGAGGTATTCGCACTGGATACGACAAAGAC |
| miR-576-5p F | GCGCGATTCTAATTTCTCCAC |
| miR-576-5p R | AGTGCAGGGTCCGAGGTATT |
| miR-609 RT primer | GTCGTATCCAGTGCAGGGTCCGAGGTATTCGCACTGGATACGACAGAGAT |
| miR-609 F | GCGCGAGGGTGTTTCTCTC |
| miR-609 R | AGTGCAGGGTCCGAGGTATT |
| miR-661 RT primer | GTCGTATCCAGTGCAGGGTCCGAGGTATTCGCACTGGATACGACACGCGC |
| miR-661 F | TGCCTGGGTCTCTGGCCT |
| miR-661 R | AGTGCAGGGTCCGAGGTATT |
| miR-1182 RT primer | GTCGTATCCAGTGCAGGGTCCGAGGTATTCGCACTGGATACGACGTCACA |
| miR-1182 F | GGAGGGTCTTGGGAGGGA |
| miR-1182 R | AGTGCAGGGTCCGAGGTATT |
| miR-1184 RT primer | GTCGTATCCAGTGCAGGGTCCGAGGTATTCGCACTGGATACGACGGAAGC |
| miR-1184 F | CGCCTGCAGCGACTTGATG |
| miR-1184 R | AGTGCAGGGTCCGAGGTATT |
